# Strategies for Optimizing the Photocatalytic Water‐Splitting Performance of Metal–Organic Framework‐Based Materials

**DOI:** 10.1002/smsc.202100060

**Published:** 2021-09-24

**Authors:** Kailai Zhang, Haijun Hu, Litong Shi, Baohua Jia, Hongwei Huang, Xiaopeng Han, Xiaodong Sun, Tianyi Ma

**Affiliations:** ^1^ Institute of Clean Energy Chemistry Key Laboratory for Green Synthesis and Preparative Chemistry of Advanced Materials College of Chemistry Liaoning University Shenyang 110036 China; ^2^ Centre for Translational Atomaterials Swinburne University of Technology Hawthorn Victoria 3122 Australia; ^3^ Beijing Key Laboratory of Materials Utilization of Nonmetallic Minerals and Solid Wastes National Laboratory of Mineral Materials School of Materials Science and Technology China University of Geosciences Beijing 100083 China; ^4^ School of Materials Science and Engineering Key Laboratory of Advanced Ceramics and Machining Technology (Ministry of Education) Tianjin Key Laboratory of Composite and Functional Materials Tianjin University Tianjin 300072 China

**Keywords:** heterojunctions, H_2_ evolution, ligand functionalizations, metal–organic frameworks, photocatalysis

## Abstract

Semiconductor photocatalytic hydrogen production technology is a pollution‐free and low‐cost technology, which is principal to alleviate the energy crisis. The metal–organic frameworks (MOFs)‐based materials have been widely used in this field because of their regular and controllable pore structure and high specific surface area. Accordingly, the recent progress of MOFs‐based materials in the production of hydrogen through water splitting is reviewed. First, the mechanism of photocatalytic hydrogen production and the existing problems are proposed and then their advantages in photocatalytic hydrogen production and various photocatalytic hydrogen production catalysts based on MOFs are summarized. Subsequently, several methods to improve their photocatalytic hydrogen production property are summarized. Finally, some views on the prospects and challenges of the use of MOFs‐based catalysts for photocatalytic hydrolysis are expounded. Although some reviews associated with MOFs for solar‐driven H_2_ production have been published, most of them focus on the classification of MOFs photocatalysts. This is the first comprehensive review concentrating on the strategies for optimizing the photocatalytic activity of MOFs‐based materials for H_2_ production. It is believed that the suggestions provided here can help to study MOFs‐based photocatalytic reactions and to further promote the development of this research direction in the future.

## Graphical abstract

Metal–organic frameworks (MOFs) possess unique pore structure, large specific surface area, modifiable skeleton, and highly crystalline nature, which can not only supply abundant reactive sites, but also extend the light response range and promote the charge carrier migration. Thus, they are widely studied in the field of photocatalysis and considered as good candidates for photocatalyzing the water reduction.

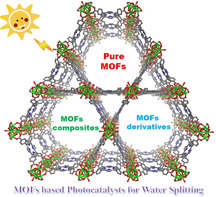

## 1 Introduction

With the explosive growth of world population and the acceleration of industrialization in the past half century, the extensive use of traditional fossil energy has not only caused many environmental problems, but also brought out energy crisis to some extent.^[^ 1, 2, 3, 4, 5, 6, 7 ^]^ It is tempting to look for a new energy source that is environmentally pollution free and can replace traditional fossil energy. Fortunately, after observing chlorophyll photosynthesis in plants, research scholars were inspired to achieve an efficient conversion of solar energy to chemical energy by designing a material like plant photosynthesis as a carrier material for artificial photosynthesis. It has been considered as one of the most important strategies to solve the future energy crisis that solar energy can be used to realize efficient solar thermal conversion and photoelectric conversion for nearly half a century.^[^ 8, 9, 10, 11, 12, 13, 14, 15 ^]^ Especially, hydrogen produced by photocatalytic decomposition of water have been considered as a new trend in the development of new energy technologies in the future.^[^ 16, 17, 18, 19, 20, 21 ^]^

**Figure 1:**
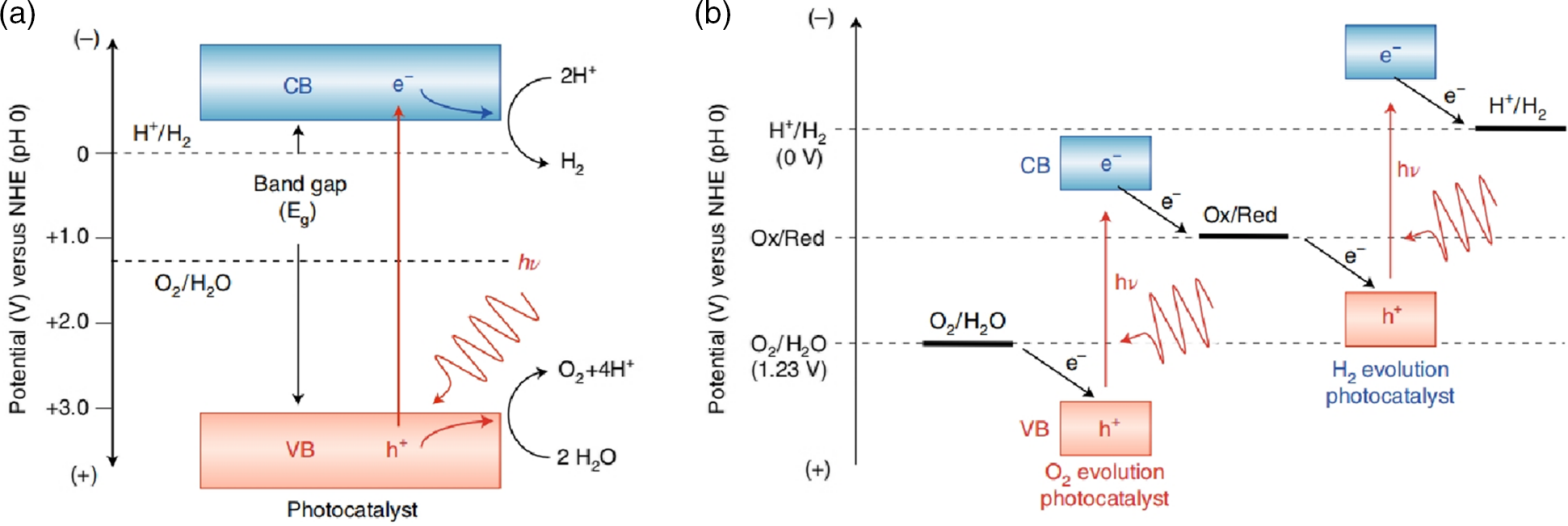
Energy diagrams for photocatalytic water splitting. a) One‐step excitation and b) two‐step excitation (Z‐scheme) processes. CB, conduction band; VB, valence band; and NHE, normal hydrogen electrode. Reproduced with permission.^[^ ^8^ ^]^ Copyright 2014, The Royal Society of Chemistry.

Usually, the water‐splitting reaction includes two half reactions, namely the oxygen evolution reaction (OER) and the hydrogen evolution reaction (HER). It is a thermodynamic climbing reaction and involves many complexities. The electronic and multistep reaction is a nonspontaneous reaction with an increase in Gibbs free energy. Under standard conditions, water is split into H_2_ and O_2_ with a ratio of 2:1, which consumes 273 kJ of energy. Therefore, it is very important to trigger the water decomposition reaction by inputting light energy into the system. The photocatalytic water‐splitting reaction converts light energy into chemical energy to produce hydrogen and oxygen. The mechanism of photocatalytic decomposition of water is as follows: first, after the semiconductor is irradiated by ultraviolet (UV)/visible light, photogenerated electrons and holes are generated. Electrons with strong reducibility are transferred from the valence band (VB) to the conduction band (CB) and react with water to reduce it into hydrogen, whereas the VB leaves strong oxidizing holes to oxidize the water into oxygen. There are two conditions for the photocatalytic water decomposition reaction to occur. One is that the minimum CB potential of the photocatalyst should be less than the redox potential of H^+^/H_2_ (0.0 V versus normal hydrogen electrode [NHE]), and the second reason is that the maximum CB potential is greater than the redox potential of O_2_/H_2_O (1.23 V versus NHE) (**Figure** **1**).^[^ ^8^ ^]^ Hence, the photocatalytic hydrogen production reaction has very high requirements on the catalyst materials. It not only needs to have a suitable energy‐level structure to absorb enough visible light and generate electron–hole pairs, but more importantly, it must effectively separate and transport photogenerated electrons and holes. At the same time, it also has efficient and stable active sites for hydrogen production. Obviously, the selection of suitable photocatalysts is very important. However, the development and application of photocatalytic technology is still limited due to several common disadvantages of photocatalysts that drive photocatalytic reactions, such as weak photocarrier separation ability, narrow full spectral response range, and fewer reactive sites. Scientists have tried to synthesize many high‐performance photocatalysts for hydrogen production reactions, among which one of the most promising materials is the metal–organic frameworks (MOFs)‐based photocatalyst.

MOFs, which are composed of metal ions and organic complexes, due to their unique porous adjustable structure and ultrahigh specific surface area, have attracted increasing attention and are widely used in various applications such as gas adsorption and separation, catalysis, energy storage, and so on.^[^ 22, 23, 24, 25, 26, 27, 28, 29, 30, 31 ^]^ In the structure of MOF‐based materials, the organic ligands can serve as antenna for adsorbing light energy, be excited, and generate the electron–hole pairs. The electrons can transfer from the ligand to the metal clusters and react with the H^+^, thus achieving effectively photocatalytic H_2_O reduction. In comparison with traditional inorganic semiconductor materials, the advantages of MOF‐based photocatalyst can be attributed to the following points. First, the unique pore structure and large specific surface area of MOF‐based materials can accelerate the migration and diffusion of reactants in MOFs and make it quickly contact the surface reactive sites and promote the reactions. Second, the bandgap of photocatalysts can be regulated by modifying organic ligands and photosensitizers with the wide light response region on their skeleton to extend the light response range. Third, the highly crystalline nature of the MOF‐based materials is beneficial for the charge carrier migration, which can improve their photocatalytic properties. Finally, their specific structure can provide convenience for researchers to study their corresponding photocatalytic reaction mechanism.^[^ 32, 33, 34, 35, 36, 37, 38 ^]^

Benefitting from the above advantages, some MOF‐based materials displayed a higher photocatalytic activity for water reduction than conventional photocatalyst materials. Although MOF‐based materials possess many advantages in photocatalytic water reduction, the stability of some MOF‐based materials is lower than conventional photocatalyst materials because of their weak coordination bond. Therefore, the structure of some MOF‐based materials is prone to be destroyed during the photocatalytic reaction, which restricts their further practical applications. Fortunately, continuous efforts have been made by researchers, and they have proposed two efficient methods to overcome the aforementioned drawbacks and construct the stable MOF‐based materials, including using high‐connected metal clusters as building units to construct MOFs and decorating hydrophobic substituents into the framework of MOF‐based materials.

Although this research topic is still in the initial stage, some efficient MOF‐based photocatalysts have been reported and exhibit excellent commendable activity for photocatalytic hydrogen production, which indicates that MOF‐based materials are good candidates for catalyzing such reactions. Therefore, a relatively comprehensive summary of strategies to improve the photocatalytic hydrogen production reaction of MOF‐based photocatalyst seems to be very forward‐looking and urgently needed. This review introduces the latest development of MOF‐based materials for photocatalytic H_2_ evolution. First, MOF‐based photocatalysts were classified. Then, the strategies to improve the hydrogen production performance of MOF‐based photocatalyst are proposed, which are mainly based on the structural diversity and tunability of MOF‐based photocatalyst, as well as their unique pore size and large specific surface area. Finally, the future application of MOF‐based photocatalyst in hydrogen production reaction is prospected and imagined. Most of previous reviews only focus on one type of MOF‐based photocatalyst, but we largely expand the scale of MOF‐based photocatalysts in our review by introducing the pure MOFs, MOFs/semiconductor composites, and all ever‐reported MOF‐derived photocatalysts for water splitting. Moreover, this review will be the first comprehensive review concentrating on the strategies for optimizing the photocatalytic activity of MOF‐based photocatalysts for H_2_ production.

## 2 The Category of MOF‐Based Photocatalysts

Thanks to the unique controllable pore structure and ultralarge specific surface area of MOF‐based photocatalysts, great breakthroughs in the field of photocatalytic hydrogen production have been witnessed in recent years. Therefore, it is advisable to comprehensively review, analyze, and summarize the series of MOF‐based hydrogen production photocatalysts.

**Figure 2:**
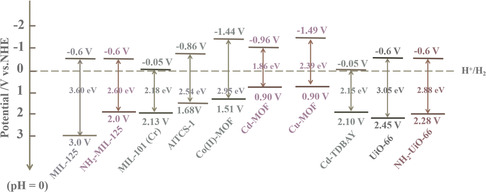
The bandgap and band position of some typical MOFs.

Then, MOF‐based photocatalysts used in hydrogen production were classified in detail, including pure MOF photocatalysts, MOF‐involved composite photocatalysts, and MOF‐derived photocatalysts. The bandgap and band position of typical MOFs are shown in **Figure** **2**.^[^ 19, 39, 40, 41, 42, 43, 44, 45 ^]^

### 2.1 Pure MOF Photocatalysts

Due to the poor separation ability of photogenerated carriers in pure MOF photocatalysts, the photocatalytic activity is limited. Therefore, there are few reports on the use of pure MOFs for the photocatalytic cracking of water. However, it is still very important in the early stages of MOF‐based photocatalysts’ development. Many metal elements on the periodic table have been attempted as metal clusters to coordinate with organic ligands to construct pure MOF photocatalysts. However, in fact, for photocatalytic hydrogen production, it is generally required that metal clusters in MOFs have a relatively variable redox state or have unsaturated metal sites as their reactive centers. Therefore, transition metal Ru^−^ and some common non‐noble metal ions with variable valence states, such as Al (III), Cu (II), Ni (II), Cd (II), Co (II), Zn (II), In (III), Cr (III), Ti (IV), and Zr (IV), have always been used to construct MOFs with photocatalytic activity for water reduction. Meanwhile, in comparison with other metals, Ti (IV) and Zr (IV) with high valence states are more suitable for constructing MOF catalysts with high photocatalytic activity for water reduction. In contrast, some metals with no variable valence state or low valence state, such as Ag^+^, Li^+^, Na^+^, and K^+^, are not suitable for constructing MOF‐based photocatalysts.

Pure MOFs photocatalysts can be classified as noble metal‐based MOF photocatalysts and non‐noble metal‐based MOF photocatalysts according to the type of metal ions or clusters in MOFs. The types of selected metal nodes have a great influence on their photocatalytic activity. On the one hand, this is because the CB position of MOF‐based catalyst is mainly contributed by the empty outer orbit of the central metal ion. Therefore, the CB position of the pure MOF photocatalysts is determined by their metal node, thus influencing their photocatalytic activity. On the other hand, in the photocatalytic hydrogen production reaction, electrons always follow a pathway from ligands to metal oxide clusters. Different metal clusters determine the separation efficiency of photogenerated carriers, which further influences their photocatalytic performance. In **Table** **1**, we show the pure MOFs composed of different central metal ions as catalysts for photocatalytic H_2_ production.

**Table 1:** Summary of pure MOF as photocatalysts for photocatalytic HER in reported publications

Photocatalysts	Metal species	Surface area [m^2^g^−1^]	Irradiation	Sacrificial agents	Photosensitizers	Cocatalysts	H_2_ production rate	Recycled times	Reference
[Al_3_(OH)_3_(HTCS)_2_]	Al	11	Visible	TEOA	–	–	50 μmol g^−1^ h^−1^	–	[39]
[Co_3_(HL)_2_·4DMF·4H_2_O	Co	343	Visible	TEOA	Ru(bpy)_3_Cl_2_	–	1102 μmol g^−1^ h^−1^	–	[40]
ZIF‐67	Co	–	Visible	TEOA	Ru(N)_3_	–	4.85 μmol h^−1^	–	[142]
ZIF‐67	Co	–	Visible	TEOA	Ru(bpy)_3_^2+^	–	843.7 μmol g^−1^ h^−1^	Five	[143]
C_21_H_21_CuN_3_O_10_S_2_	Cu	–	Near‐infrared	TEOA	–	Pt	18.96 μmol h^−1^	Five	[41]
[Cu_2_I_2_(BPEA)](DMF)	Cu	–	UV−vis	Methanol	–	Pt	32 μmol g^−1^ h^−1^	Three	[50]
[Cu(RSH)(H_2_O)]_*n*_	Cu	–	Visible	TEOA	Eosin Y		7.88 mmol g^−1^ h^−1^	Four	[144]
Cu‐I‐bpy	Cu	–	UV	TEA	–	–	7.09 mmol g^−1^ h^−1^	Five	[145]
Cd‐TBAPy	Cd	27	Visible	TEOA	–	Pt	4.3 μmol h^−1^	–	[42]
UiO‐66	Zr	972	UV−vis	Methanol	–	Pt	0.8 mL h^−1^	–	[43, 44]
UiO‐66‐NH_2_	Zr	789	UV−vis	Methanol	–	Pt	0.93 mL h^−1^	–	[44, 45]
[Ru_2_(p‐BDC)_2_]_*n*_	Ru	516.5	Visible	EDTA‐2Na	Ru(bpy)_3_^2+^	–	16.1 μmol h^−1^	–	[46]
[Ru_2_(p‐BDC)_2_]_*n*_	Ru	474.7	Visible	EDTA‐2Na	Ru(bpy)_3_^2+^	–	13.8 μmol h^−1^	–	[47]
[Ru_2_(p‐BDC)_2_Br]_*n*_	Ru	379.3	Visible	EDTA‐2Na	Ru(bpy)_3_^2+^	–	11.7 μmol h^−1^	–	[48]
[Ru_2_(ZnTCPP)BF_4_]_*n*_	Ru	413.9	UV−vis	EDTA‐2Na	–	–	TON_24h_ = 29.9	–	[49]
Ru‐TBP‐Zn	Ru	422	Visible	TEOA	–	–	0.24 mmol g^−1^ h^−1^	Six	[146]
[Ni_2_(PymS)_4_]	Ni	–	Visible	TEA	Fluorescein	–	TOF = 10.6 h^−1^	Four	[51]
Ti‐MOF‐Ru(tpy)_2_	Ti	20	Visible	TEOA		Pt	1.82 μmol h^−1^	Three	[52]
MIL‐167	Ti	–	UV−vis	TEA	–	–	7.7 μmol h^−1^	–	[53]
[Zn{Pd(INA)_4_}]_*n*_	Zn	14.1	Visible	EDTA‐2Na	Ru(bpy)_3_^2+^	–	TON_4h_ = 28.2	–	[147]
[Zn_2_(H_2_O)_3_{PdCl_2_(pydc)_2_}]_*n*_	Zn	47.2	Visible	EDTA‐2Na	Ru(bpy)_3_^2+^		TON_4h_ = 20.2	–	[148]
MIL‐101	Cr	–	Visible	TEOA	Erythrosin B dye	Ni/NiO_x_	125 μmol h^−1^	–	[149]

#### 2.1.1 Noble Metal‐Based MOF Photocatalysts

Precious metals easily adsorb reactants on their surface during the reaction process because their *d*‐electron orbitals are not filled. Meanwhile, because of their excellent properties such as high temperature resistance, oxidation resistance, and corrosion resistance, the precious metal‐based MOF photocatalyst has strong photocatalytic activity. The first application of precious metal‐based MOF photocatalysts in water decomposition reaction was reported by Mori and coworkers.^[^ ^46^ ^]^ They created new types of porous Ru‐MOFs, named [Ru_2_(p‐BDC)_2_]_*n*_, which showed remarkable feats of photocatalytic hydrogen production performance in reaction solution containing EDTA‐2Na, Ru(bpy)_3_Cl_2_, MV^2+^, and Ru^−^ photocatalyst. After 4 h illumination, the turnover number (TON) can reach up to 8.16 and 81.6 based on [Ru_2_(p‐BDC)_2_] and Ru(bpy)_3_^2+^_*n*_, respectively. A series of similar works from Mori subsequently confirmed the potential advantage of Ru‐based MOFs for photocatalytic hydrogen production.^[^ 47, 48, 49 ^]^ Although noble metal‐based MOF photocatalysts all showed excellent photocatalytic hydrogen production efficiency, they were not commonly used in photocatalytic water decomposition reaction due to their high cost. Therefore, researchers turned to search for non‐noble metal‐based MOF catalysts with low cost. Non‐noble metal‐based MOF catalysts mainly include transition metal‐based MOF photocatalysts.

#### 2.1.2 Transition Metal‐Based MOF Photocatalysts

**Figure 3:**
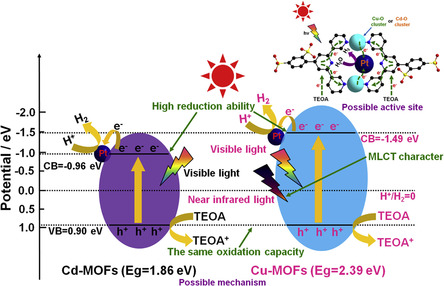
A possible active site and mechanism for the photocatalytic H_2_ evolution of Cd‐MOFs and Cu‐MOFs. Reproduced with permission.^[^ ^41^ ^]^ Copyright 2017, Elsevier.

Transition metal‐based MOF photocatalysts have been extensively studied because transition metals, as the core metals, are abundant in the Earth and are easily obtained. Moreover, they also have the advantage of relatively excellent catalytic activity compared with precious metals. For example, Lan's group synthesized two kinds of Al‐based MOFs (Al‐MOFs), among which the AlTCS‐1 displayed an excellent photocatalytic hydrogenation performance under visible light irradiation, and the average hydrogen production rate can reach up to 50 μmol g^−1 ^h^−1^.^[^ ^39^ ^]^ In addition to Al‐MOFs, Cu‐MOFs are also widely used in photocatalytic water reduction. Copper is one of the earliest metals used by mankind. Its content on the Earth is relatively abundant and the mining cost is low. For Cu‐based MOFs, the central divalent copper ion is usually four coordinated or six coordinated with unsaturated coordination sites, which can be regarded as reactive sites for promoting the photocatalytic reaction. For example, Cheng and coworkers prepared a novel 2D Cu(I)‐based MOF material, which showed high catalytic hydrogen production performance under solar light.^[^ ^50^ ^]^ The high photocatalytic hydrogen production activity was mainly attributed to the presence of Cu(I) center, which can serve as an electron transfer intermediate to improve the charge transfer efficiency. In addition, Zeng and coworkers used 4‐(2,4‐disulfophenyl)‐3,2:6,3 terpyridine (H_2_DSPTP) to coordinate with Cd and Cu ions to form two novel Cd‐MOFs and Cu‐MOFs and determined their band positions.^[^ ^41^ ^]^ It was found that the CB position of Cu‐MOFs was lower than that of Cd‐MOFs. It is interesting that although they showed good activity for photocatalytic hydrogen production under visible light conditions, only Cu‐MOFs with suitable bandgap showed hydrogen production activity under infrared light irradiation. This issue explained that appropriate band design for MOF‐based catalyst is of great practical significance (**Figure** **3**).

In addition to aforementioned Al‐ and Cu‐based MOFs, other transition metal‐based MOF photocatalysts have also been reported in recent years. Su and coworkers successfully constructed amino‐functionalized Co(II)‐MOFs with semiconductor nature through one‐pot solvothermal method.^[^ ^40^ ^]^

**Figure 4:**
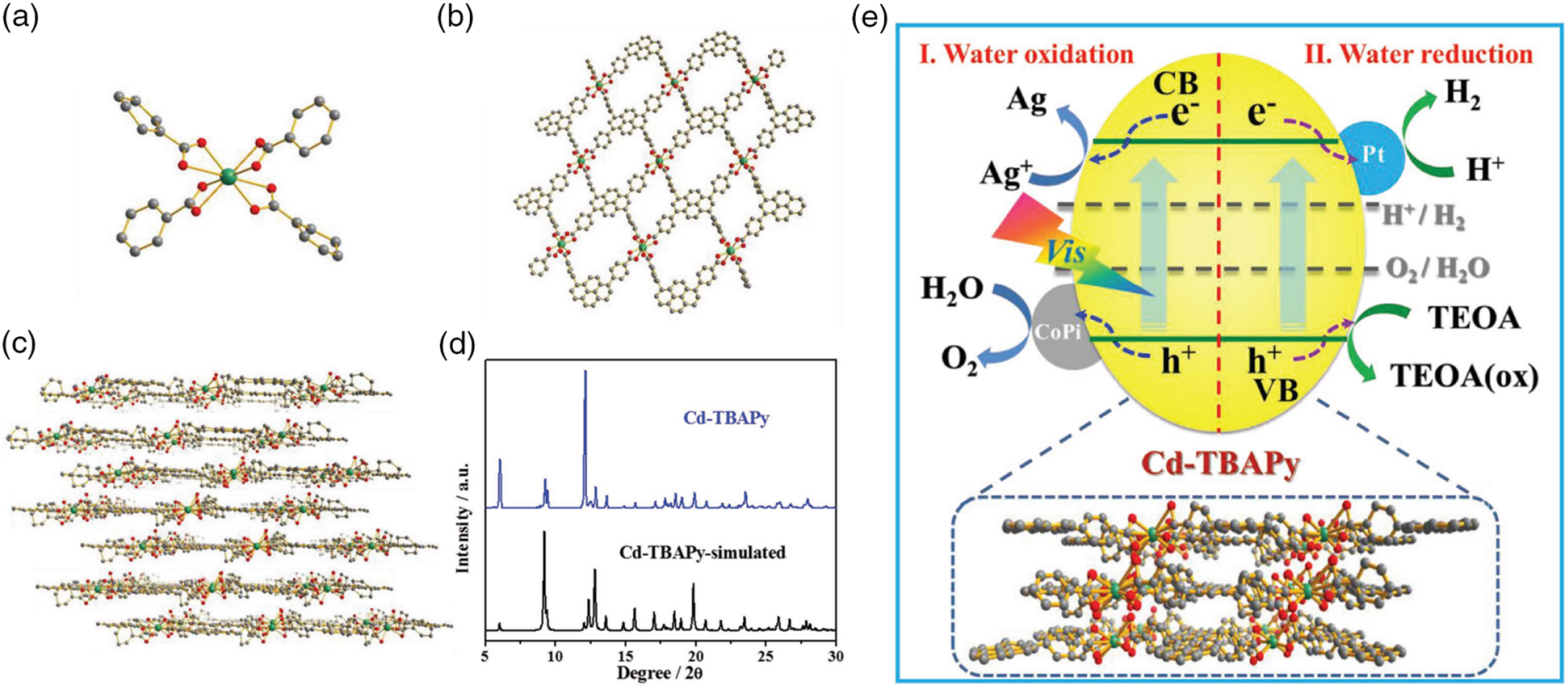
The structure and analysis of Cd‐TBAPy: a) the coordination mode of the Cd atom in Cd‐TBAPy, b) the single‐layer structure of Cd‐TBAPy viewed along the *b*‐axis, c) the 2D layered structure of Cd‐TBAPy, and d) X‐ray diffraction (XRD) patterns of Cd‐TBAPy and Cd‐TBAPy simulated. Color representation: red, O; gray, C; and green, Cd. H atoms are removed for clarity. e) The proposed mechanism for visible light‐driven photocatalytic H_2_ and O_2_ evolution over Cd‐TBAPy. a–e) Reproduced with permission.^[^ ^42^ ^]^ Copyright 2018, Wiley‐VCH.

It was the first example of trinuclear Co‐MOFs being applied in the field of photocatalytic hydrogen evolution. By regulating the experimental conditions such as photosensitizer, TEOA, solvents, and size of catalysts, such Co‐based MOFs displayed high photocatalytic activity for H_2_ production, in which the H_2_ production rate was as high as 1102 μmol g^−1^ h^−1^. Moreover, Li and coworkers reacted 1,3,6,8‐tetrabenzoic acid‐pyrene (TBAPy) agent with Cd^2+^ to construct a novel bifunctional Cd‐TBAPy and used it to catalyze the decomposition of water to produce H_2_ and O_2_ under sunlight.^[^ ^42^ ^]^ The Cd atom is coordinated with eight oxygen atoms from four different ligands using a Cd—O bond length of about 2.348 Å. Moreover, the backbone of Cd‐TBAPy presented a 2D eclipsed structure due to the coordination effect, in which all single layers are sequentially stacked with an interlayer spacing of about 4.28 Å. Moreover, the prepared Cd‐MOFs had high purity and crystallinity. This is the first example where MOF‐based photocatalysts have been used for achieving both photocatalytic hydrogen and oxygen production reactions, providing a new idea for designing MOF‐based photocatalysts for water splitting (**Figure** **4**).

Moreover, Du's group designed a 2D Ni‐MOF [Ni_2_(Pyms)_4_] photocatalyst for hydrogen production.^[^ ^51^ ^]^ In their work, the effects of particle size, original pH value, electron donor, and dye on the photocatalytic hydrolysis efficiency were investigated. The authors confirmed the oxidative quenching process by cyclic voltammograms (CV) and fluorescence spectrum. At the same time, the mechanism of the hydrogen production cycle was systematically studied by photochemical analysis.

Although transition metal‐based MOFs have been widely studied, due to the reversible nature of coordination bonds, most of transition metal‐based MOFs lack stability and are not stable in water systems compared with other conventional photocatalyst materials, which restricted their practicability. To overcome this drawback, Zr‐based MOFs, whose coordination bonds have the strength of covalent bonds, and exhibit excellent chemical stability, have been widely studied and have been considered as good candidates for promoting photocatalytic water reduction. UiO‐66 is one of the most widely studied Zr‐based MOFs, whose photocatalytic activity for hydrogen production was first reported and studied by Garcia and coworkers.^[^ ^44^ ^]^ It was found that when amino and precious metal platinum (Pt) were introduced into the material, a significant increase in photocatalytic activity was witnessed. Since then, reports on Zr‐based MOFs, used in photocatalytic hydrogen production, have gradually increased. However, due to the relatively weak oxidation performance of Zr‐based MOFs, people gradually shifted their attention to the Ti‐based MOF photocatalyst with stronger oxidation capacity. For example, Matsuoka and coworkers studied the photocatalytic decomposition of water by Ti‐MOF‐Ru(TPY)_2_.^[^ ^52^ ^]^ Ti‐MOF‐Ru(TPY)_2_ was assembled by the Ti clusters and bis(4′‐(4‐carboxyphenyl)‐terpyridine)Ru(II) (Ru(TPY)_2_) ligand, which can extend the light absorption region to 620 nm. The introduction of bis (4′‐(4‐carboxyphenyl)‐terpyridine) Ru(II) (Ru(TPY)_2_) can affect the photoelectron transfer process, which may be attributed to its wider photoresponse range. Then, Devic et al. synthesized four kinds of Ti‐MOFs materials, including MIL‐167, MIL‐168, MIL‐169, and “NTU‐9‐like,” through the coordination assembly of 2,5‐dihydroxyterephthalic acid (H_4_DOBDC) and Ti ions. Benefitting from the strong redox activity and excellent light response performance, all constructed Ti‐MOFs displayed commendable photocatalytic activity for hydrogen production.^[^ ^53^ ^]^

### 2.2 MOF‐Involved Composite Photocatalysts

MOF‐involved composite photocatalysts could take the advantages of unique porosity and a large Brunner‐Emmet‐Teller (BET) specific surface area of MOF materials; thus, some catalytic active materials, including nanoparticles (NPs) and photocatalytic active composites, can be loaded into their pores. MOFs and these active materials have synergistic effects, thus effectively promoting water cracking to produce H_2._ The Pt NPs are often used as cocatalysts to improve the photocatalytic performance of the main catalyst. By injecting a small amount of Pt into the MOF structure, the separation ability of photogenerated carriers can be improved, and the photocatalytic activity of the MOF‐based photocatalyst can be significantly improved. Jiang et al. embedded Pt NPs with a diameter of about 3 nm into amino‐modified UiO‐66 and then evaluated its photocatalytic hydrogen production activity.^[^ ^54^ ^]^ The author pointed out that the presence or absence of Pt NPs greatly affected their photocatalytic efficiency because the existence of Pt NPs can shorten the transfer distance of photogenerated carriers and improve their separation efficiency. Besides Pt, other precious metals, such as Ag, can also be incorporated with MOF materials to enhance their photocatalytic activity. Yang and coworkers used dye‐like azo‐carboxylic acid as an organic linker to build a Gd^3+^‐based MOF: named [Gd_2_(abtc)(H_2_O)_2_(OH)_2_]2H_2_O (Gd‐MOF) (H_4_abtc = 3,3′,5,5′‐azobenzene tetracarboxylic acid).^[^ ^55^ ^]^ The synthesized catalysts exhibited super excellent activity for HER. The results proved that the hydrogen production activity was closely related to the amount of Ag. The author confirmed that the noble metal Ag on the MOF surface has the plasmon resonance effect through impedance analysis and photoluminescence (PL) spectroscopy, which can effectively enhance the light‐harvesting performance and promote the photogenerated carrier separation ability of the MOF‐based catalysts.

Apart from precious metals, Ni NPs have also been regarded as excellent cocatalysts for promoting charge migration. For example, Lu et al. reported a Ni (111) NPs‐deposited MOF‐5 based‐photocatalyst, named Ni@MOF‐5, which was used for hydrolysis of water to produce hydrogen.^[^ ^56^ ^]^

**Figure 5:**
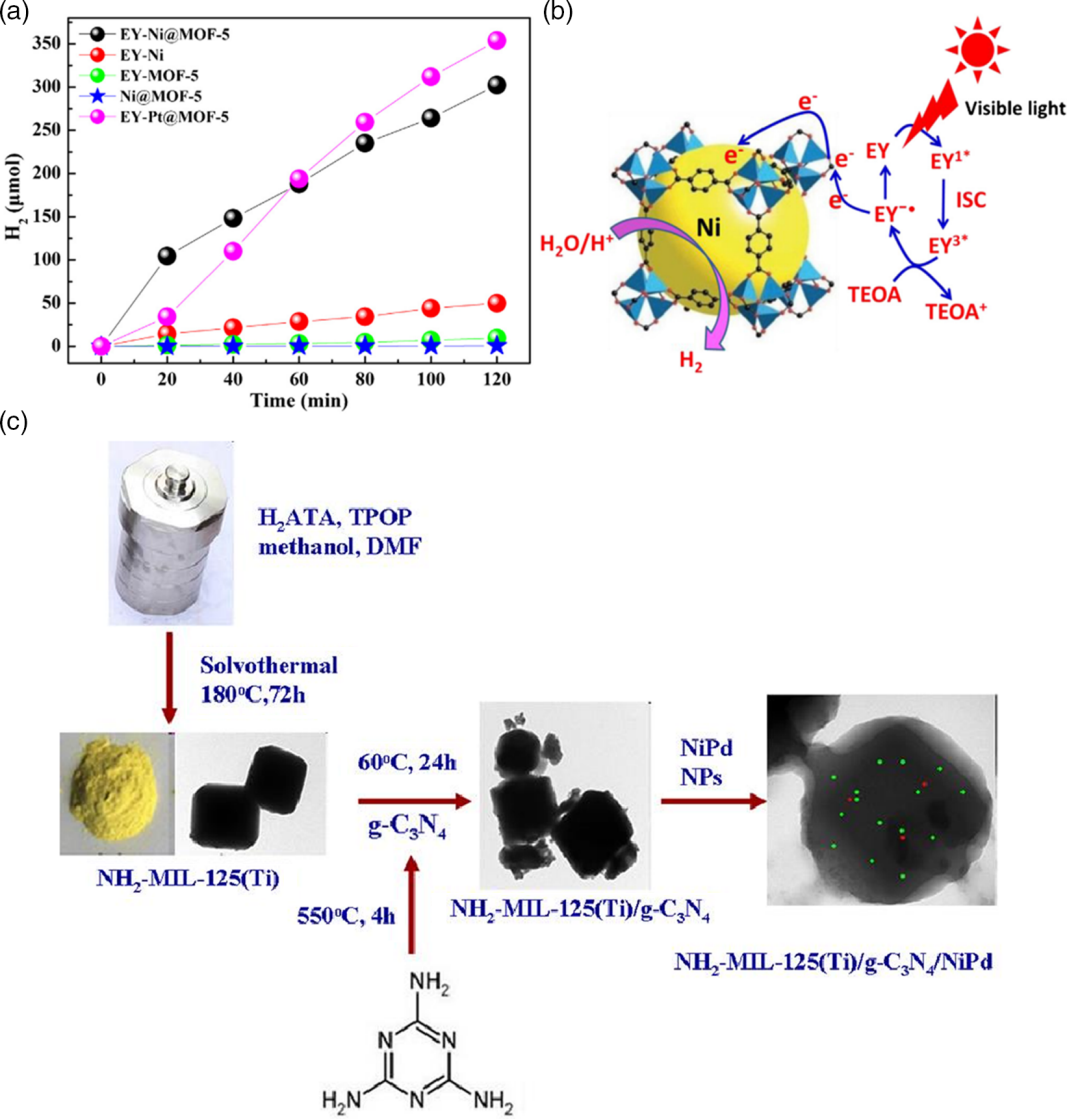
a) The time courses of hydrogen evolution over EY‐Ni, EY‐MOF‐5, EY‐Ni@MOF‐5, EY‐Pt@MOF‐5, and Ni@MOF‐5 photocatalysts in 100 mL 10% (v/v) TEOA aqueous solution (pH = 11) under visible light irradiation (*λ* ≥ 420 nm). b) Photocatalytic mechanism for H_2_ evolution over the EY‐sensitized Ni@MOF‐5 with TEOA as a sacrificial donor under visible light irradiation. a,b) Reproduced with permission.^[^ ^56^ ^]^ Copyright 2016, Elsevier. c) Illustration of the preparation of NH2‐MIL‐125(Ti)/CN/NiPd photocatalyst. Reproduced with permission.^[^ ^57^ ^]^ Copyright 2017, Elsevier.

Through the activity comparison test under the same conditions, its scalar quantum efficiency can reach up to 16.7% after 2 h of visible light irradiation. They found that in the photocatalytic hydrogen production reaction, electrons can be transferred from Eosin Y (EY) to the active sites of Ni (111) NPs to accelerate the reaction (**Figure** **5**a). Furthermore, Wang's group used Ni–Pd cocatalyst and carbon nitride to modify NH_2_‐MIL‐125(Ti) through a facile method (Figure 5b).^[^ ^57^ ^]^ The Ni–Pd cocatalyst embedded on it can significantly promote the photocatalytic activity of NH_2_‐MIL‐125(Ti)/g‐C_3_N_4._ The supported metal NPs could not only enhance the responsiveness of the catalyst to visible light, and subsequently generate more photogenerated carriers, but also promote the transfer of electrons to the CB of the semiconductor. The optimal hydrogen production efficiency can reach up to 8.7 mmol g^−1^ h^−1^, which is 322 and 1.3 times higher than those of NH_2_‐MIL‐125(Ti)/0.75CN and NH_2_‐MIL‐125(Ti)/Ni_15.8_Pd_4.1_, respectively.

**Figure 6:**
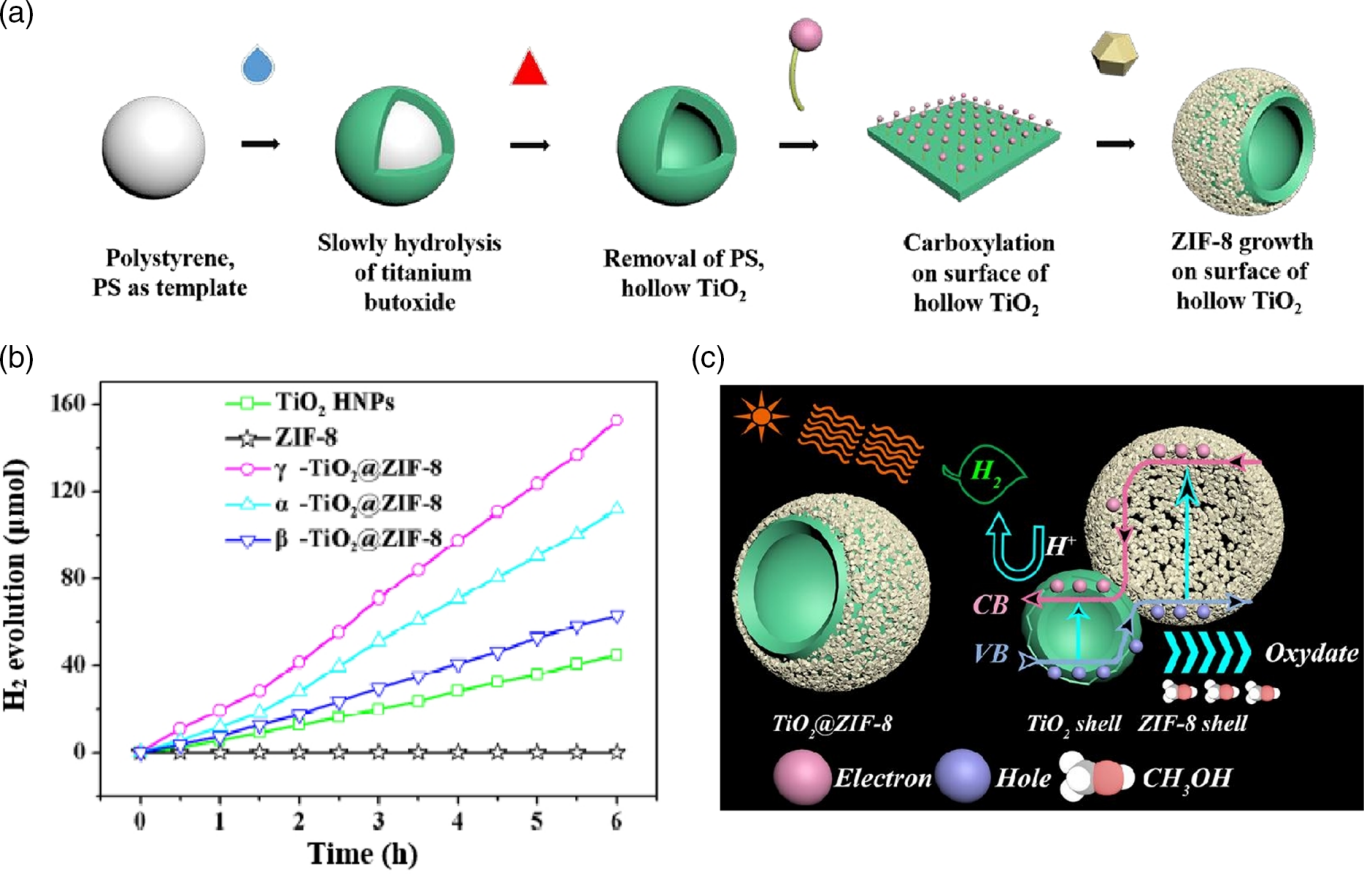
a) Schematic illustration of the integration of double‐shell TiO_2_@ZIF‐8 HNP. b) Photocatalytic hydrogen evolution performance of TiO_2_ HNPs, ZIF‐8, and α‐/β‐/γ‐TiO2@ZIF‐8. c) Mechanism of H_2_ evolution reaction over TiO_2_@ZIF‐8. a–c) Reproduced with permission.^[^ ^61^ ^]^ Copyright 2019, Elsevier.

In addition to Ni NPs, some metal sulfides or metal oxides, such as MoS_2_, CdS, and TiO_2_ NPs, are also often be used to modify MOF‐based photocatalysts to improve their photocatalytic activity. Ma et al. used MoS_2_ NPs to modify zeolitic imidazolate framework (ZIF)‐67 doped with carbon nitride. Through a series of photoelectric tests, they confirmed that the addition of MoS_2_ can broaden the visible light response ability and improve the separation capability of photogenerated electrons and holes.^[^ ^58^ ^]^ Later, Stylianou and coworkers synthesized Mo_3_S_13_^2−^ nanocluster and 1 T MoS_2_ NPs‐modified NH_2_‐MIL‐125 for photosplitting water to produce hydrogen.^[^ ^59^ ^]^ With Mo_3_S_13_^2−^ nanocluster and 1 T MoS_2_ NPs as promoters, the prepared composite catalyst had strong photocatalytic activity. The hydrogen production rate can reach up to 2094 and 1454 mmol g^−1 ^h^−1^ for Mo_3_S_13_^2−^ nanoclusters and 1 T MoS_2_ NPs, respectively. In virtue of the high surface area, MOF‐based catalysts are often used to integrate with CdS to improve the light response range and restrict the charge carrier recombination. Through experimental research, Sun's group found that embedding CdS into MOFs can significantly enhance the efficiency of photocatalytic hydrogen production.^[^ ^60^ ^]^ In this composite photocatalyst, MIL‐101 can provide stronger adsorption sites and photocatalytic reaction centers. In contrast, CdS can extend its light absorption region, thus endowing it with excellent photocatalytic activity for water splitting. Moreover, Pan and coworkers modified ZIF‐8 on titanium dioxide hollow nanospheres (TiO_2_ HNPs) through a facile sonochemical route.^[^ ^61^ ^]^ Due to the efficient charge separation ability confirmed by the characterization of PL, time‐resolved PL (TRPL) decay transient, electrochemical impedance spectra (EIS), and photocurrent (PC) response, the electrons can be easily injected from ZIF‐8 to TiO_2_ HNPs and the powerful active sites provided by ZIF‐8. Consequently, the catalyst displayed excellent photocatalytic activity for hydrogen production, and the apparent quantum efficiency (AQE) can reach up to 50.89% under simulated visible light (**Figure** **6**).

Yuan and coworkers used a covalently bonded Fe_2_S_2_ catalyst to assemble with UiO‐MOFs, forming the UiO‐MOF‐Fe_2_S_2_ material.^[^ ^62^ ^]^ This composite can not only enhance their stability, but also improve the electron transfer efficiency between Fe_2_S_2_ and UiO‐MOFs. The excellent results showed that the use of Fe_2_S_2_ complex to modify MOFs was an ideal strategy to improve their visible light catalytic performance.

**Figure 7:**
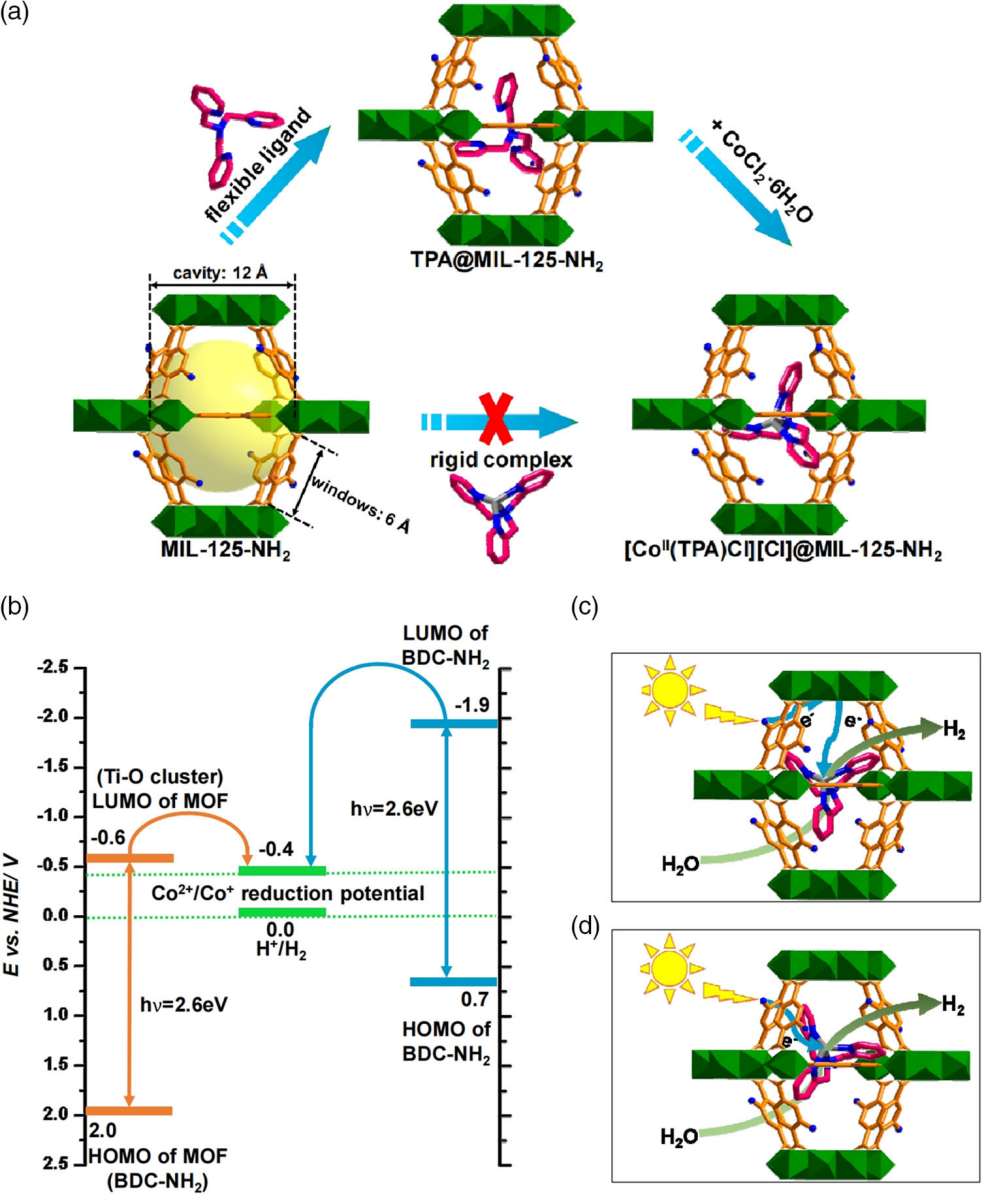
a) Schematic illustration of the “ship‐in‐a‐bottle” synthesis of Co(II)@MIL‐125‐NH_2_. b) Schematic diagram of redox potentials of MIL‐125‐NH_2_, BDC‐NH_2_, the Co(II) complex, and H^+^/H_2_. Proposed electron transfer route c) between MIL‐125‐NH_2_ and the Co(II) complex and d) between BDC‐NH_2_ and the Co(II) complex. a–d) Reproduced with permission.^[^ ^64^ ^]^ Copyright 2016, American Chemical Society.

Moreover, Gascon and coworkers assembled a highly photoactive cobaloxime catalyst into NH_2_‐MIL‐125(Ti) through a special “ship‐in‐the‐bottle” strategy.^[^ ^63^ ^]^ The synthesized Co@MOFs exhibited excellent photocatalytic hydrogen production activity with NH_2_‐MIL‐125(Ti) as photosensitizer and TEA as sacrificial agent. This is a typical example of using a non‐noble metal Co@MOF catalyst as a high‐performance MOF‐based multifunctional catalyst. Recently, Jiang's group used a similar synthesis strategy to assemble Co(II) molecular [CoII(TPA)Cl][Cl] (TPA = tris(2‐pyridylmethyl)amine) into the MIL‐125‐NH_2_ photosensitizer (**Figure** **7**).^[^ ^64^ ^]^ The synthesized composite catalyst can significantly promote the water‐splitting efficiency. In this photocatalytic system, the Co(II) molecule can serve as electron acceptor, thus making a contribution to impeding the electron–hole pair recombination. Therefore, with TEOA as the sacrificial agent, such a Co(II) molecule‐modified Ti‐MOF‐based photocatalyst exhibited a commendable property for photocatalytic water reduction, in which the largest hydrogen production rate could reach 553 μmol g^−1 ^h^−1^.

### 2.3 MOF‐Derived Photocatalysts

Benefitting from the large BET surface area and ordered structure, MOF materials can also serve as a template to construct MOF‐derived photocatalysts, including CdS, TiO_2_, C/N, composites and so on. In comparison with original materials, the photocatalysts constructed from the MOF‐based template possess not only specific topography and facet but also high specific surface area. Therefore, such kinds of MOF‐derived photocatalysts have been widely studied in the field of photocatalytic water reduction in recent years. In **Table** **2**, we show the examples of MOF‐derived photocatalysts for H_2_ production.

**Table 2:** A summary of MOF‐derived photocatalysts for photocatalytic HER in reported publications

Photocatalysts	Surface area (m^2^g^−1^)	Template of MOFs	Irradiation	Sacrificial agents	Cocatalysts	H_2_ production rate	Recycled times	Reference
Co_4_S_3_/CdS	24.5	Co‐MOF	Visible	Lactic acid	–	5892.6 μmol g^−1^ h^−1^	Four	[65]
NH_2_‐MIL‐125/TiO_2_/CdS	636	NH_2_‐MIL‐125	Visible	Na_2_S/Na_2_SO_3_	Pt	2997.482 μmol g^−1^ h^−1^	Three	[66]
Zn_0.5_Cd0_.5_S/M‐TiO_2_	105	MIL‐125‐NH_2_ (Ti)	Visible	Na_2_S/Na_2_SO_3_	–	180.4 mmol g^−1^ h^−1^	Four	[67]
N‐doped graphene	874.5	ZIF‐8	Visible	TEOA	Pt	18.5 μmol h^−1^	–	[68]
C‐ZrO_2_/g‐C_3_N_4_/Ni_2_P	–	UiO‐66‐NH_2_/g‐C_3_N_4_	Visible	TEOA	Ni_2_P	10.04 mmol g^−1^ h^−1^	Three	[69]
HP‐CdS	119	MOF‐53(Al)	Visible	Na_2_S/Na_2_SO_3_	–	630.4 μmol g^−1^ h^−1^	Four	[150]
CdS‐YS	94	Cd_3_[Fe(CN)_6_]_2_	Visible	Na_2_S/Na_2_SO_3_	–	3051.4 μmol g^−1^ h^−1^	Four	[151]
CdS/ZCO	–	ZnCo‐ZIF	Visible	Lactic acid	–	3978.6 μmol g^−1^ h^−1^	Four	[152]
CdS/MCTMPs	–	ZIF‐67	Sunlight	Lactic acid	–	136.77 μmol mg^−1^ h^−1^	Five	[153]
Ni_2_P/CdS	–	Ni‐BTC	Sunlight	Lactic acid	Ni_2_P	33 480 μmol g^−1^ h^−1^	Five	[154]
ZrO_2_/g‐C_3_N_4_/Ni_2_P	–	NH NH_2_‐UiO‐66/g‐C_3_N_4_	Visible	TEOA	Ni_2_P	10.04 mmol g^−1^ h^−1^	Four	[155]
Fe_2_O_3_@TiO_2_	11.6	MIL‐101(Fe)	Visible	TEA	Pt	0.625 μmol mg^−1^ h^−1^	Three	[156]
MoS_2_@TiO_2_	–	NH_2_‐MIL‐125	Visible	TEOA	–	10 046 μmol g^−1^ h^−1^	Three	[157]
Cu/TiO_2_‐AA	–	Cu_3_(BTC)_2_@TiO_2_	UV	Methanol	–	62.16 μmol g^−1^ h^−1^	–	[158]
FeO_3.3_C_0.2_H_1.0_	93.7	MIL‐101(Fe)	Visible	TEA	Pt	28.3 μmol h^−1^	Four	[159]
Co_3_O_4_/TiO_2_	30.16	Co‐MOF	UV−vis	Methanol	–	6.4 mmol g^−1^ h^−1^	–	[160]

For example, Lv's group synthesized Co_4_S_3_‐decorated CdS photocatalyst through a facile method using 2D MOFs as sacrificial templates.^[^ ^65^ ^]^ Compared with pure CdS nanospheres, the MOF‐derived photocatalysts not only exhibited a higher surface area, but can also provide more reactive sites.

More importantly, the maximum light absorption wavelength of the composite can be broadened from 570 to 720 nm. The improved light adsorption can make contributions to promoting the activity for photocatalytic water reduction, in which the H_2_ evolution rate can reach up to 5892.6 μmol g^−1^ h^−1^, six times higher than that of bare CdS. Apart from CdS, TiO_2_‐based photocatalysts with porous structures have also been widely constructed using MOF materials as templates. A remarkable work was reported by Zhou's group.^[^ ^66^ ^]^ In their work, two novel visible light‐active NH_2_‐MIL‐125/TiO_2_/CdS yolk–shell and hollow H‐TiO_2_/CdS hybrid heterostructure catalysts were successfully constructed via the hydrolysis of NH_2_‐MIL‐125, thioacetamide (CH_3_CSNH_2_), and cadmium acetate (Cd (CH_3_COO)_2_·2H_2_O) through a postsolvothermal method. The above two photocatalysts both displayed excellent performance for H_2_ evolution, with the AQE of 4.81% and 2.41% at 420 nm, respectively. These excellent results can be ascribed to the porous structure, abundant reactive sites, and intimate contact, provided by the MOF materials. Subsequently, Chen and coworkers designed a series of crown‐like Zn_0.5_Cd_0.5_S MTiO_2_ composites derived from MOFs.^[^ ^67^ ^]^

**Figure 8:**
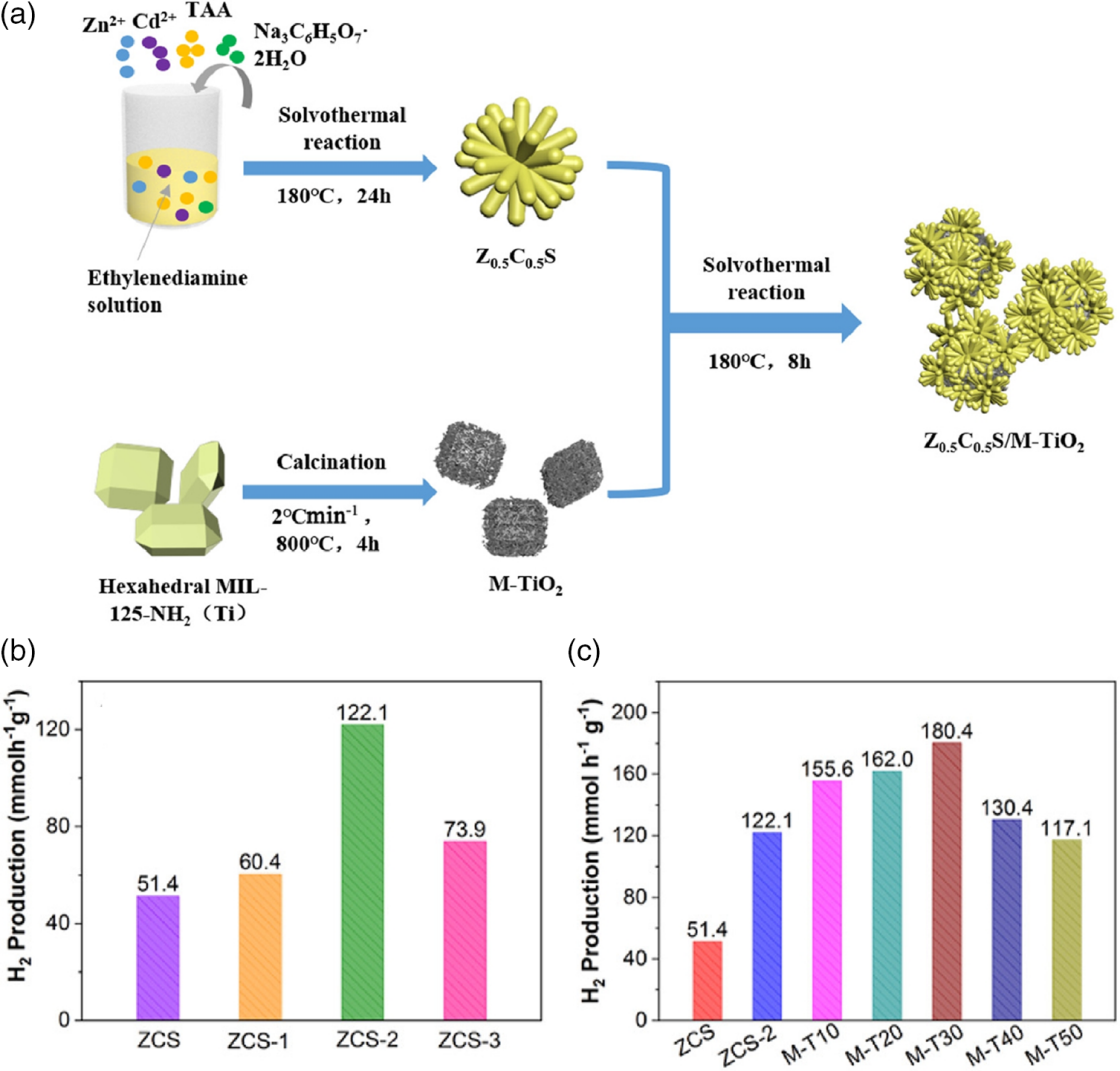
a) Schematic illustration of the preparation processes of Zn_0.5_Cd_0.5_S/M‐TiO_2_ photocatalyst. b) Hydrogen production performance of ZCS materials. c) ZCS‐2, M‐TiO_2_, and different ratios of ZCS‐2/M‐TiO_2_ composites. a–c) Reproduced with permission.^[^ ^67^ ^]^ Copyright 2021, Elsevier.

Compared with pure Zn_0.5_Cd_0.5_S or M‐TiO_2_, the AQE of the optimal amount Zn_0.5_Cd_0.5_S MTiO_2_ composite at 420 nm can reach up to 48.9% and the hydrogen production rate can reach to 180.4 mmol g^−1 ^h^−1^. The huge improvement in photocatalytic activity was mainly due to the enlarged specific surface area, more exposed active sites, and the enhancement of the electron–hole separation efficiency (**Figure** **8**).

**Figure 9:**
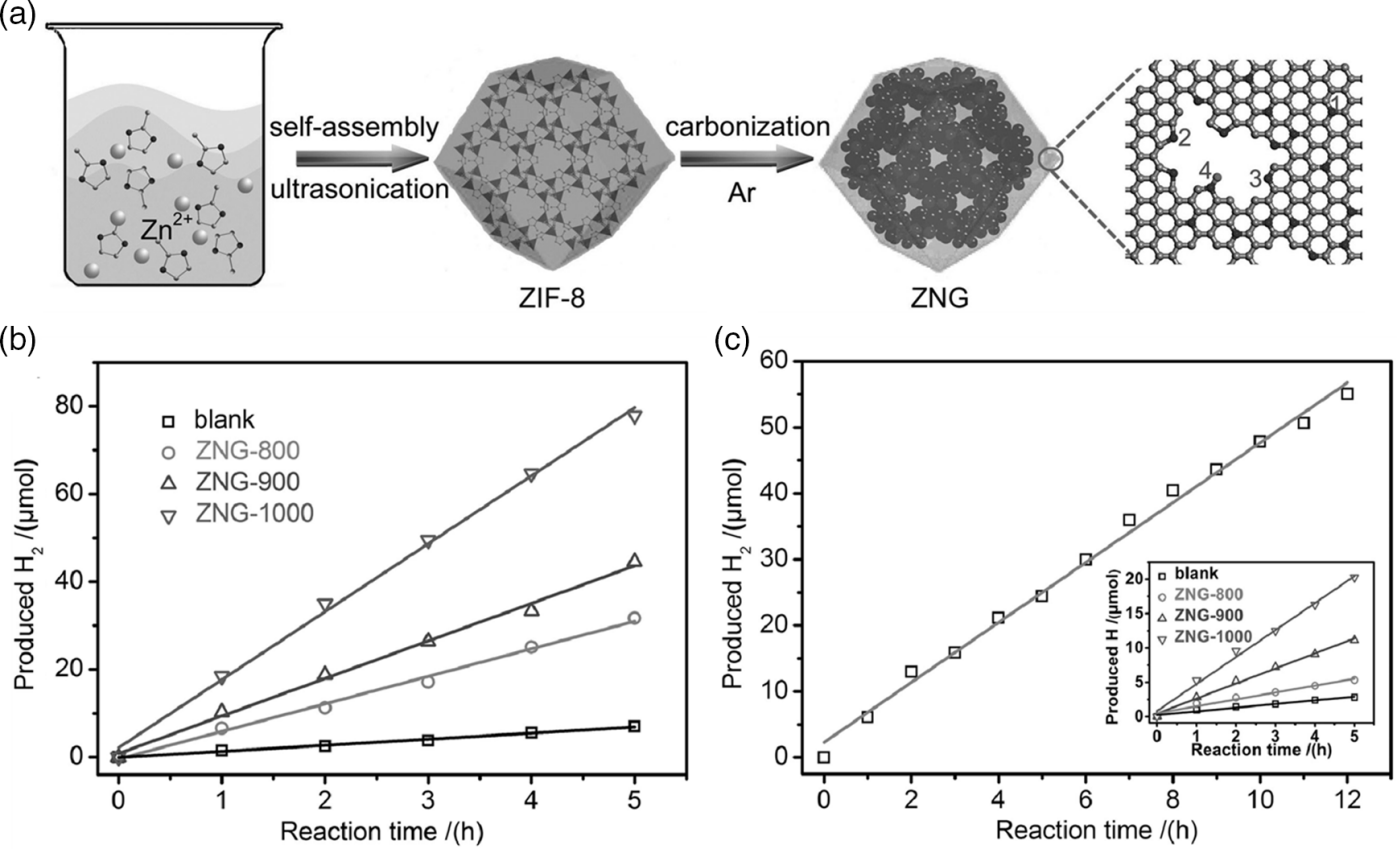
a) Schematic illustration of the synthetic procedure of ZNGs and four types of bonding configurations of N atoms (1 graphitic *N*, 2 pyrrolic *N*, 3 pyridinic *N*, and 4 pyridine‐*N*‐oxide). b) Photocatalytic activities of ZNGs for H_2_ production with Pt as a cocatalyst. c) Photocatalytic activity of ZNG‐1000 under a prolonged light illumination duration of 12 h without Pt as a cocatalyst (inset: time course of the H_2_ evolution rate of ZNGs without Pt as a cocatalyst). a–c) Reproduced with permission.^[^ ^68^ ^]^ Copyright 2017, Wiley‐VCH.

In addition, C/N composites designed using MOFs as template can also be regarded as an efficient photocatalytic material to drive hydrogen production reaction. For instance, Cheng's group reported N‐doped graphene prepared using nitrogen‐rich MOF‐ZIF‐8 as a template.^[^ ^68^ ^]^ Utilizing Pt and triethanolamine as cocatalyst and hole scavenger, respectively, ZNG‐1000 displayed the highest catalytic hydrogen production rate under visible light, up to 18.5 μmol h^−1^ (**Figure** **9**b–c). The high content of graphite‐phase carbon nitride can prevent the recombination of photogenerated carriers and ultimately enhance the photocatalytic activity **(**Figure 9 **)**. Subsequently, a C‐doped ZrO_2_/g‐C_3_N_4_/Ni_2_P (C‐ZrO_2_/g‐C_3_N_4_/Ni_2_P) ternary composite was successfully fabricated by a simple and feasible self‐assembly process combined with single‐step annealing treatment, and then the application of the photocatalytic decomposition of water to produce hydrogen was realized.^[^ ^69^ ^]^ Closely bonded heterojunction endowed the catalytic materials with efficient space separation ability of photogenerated carriers between C‐ZrO_2_ and g‐C_3_N_4_. Encouragingly, this heterojunction showed outstanding performance for photocatalytic H_2_ production, in which the hydrogen production rate could reach 10.04 mmol g^−1^ h^−1^, and the AQE can reach 35.5% at 420 nm.

In brief, using MOF‐derived materials to conduct the photocatalytic hydrogen production reaction is very beneficial to enhance the performance. The obtained materials can inherit the ultrahigh specific surface area and large porosity of MOF materials. Therefore, in comparison with pristine photocatalysts, the obtained MOF‐derived photocatalysts, which can provide more reactive sites, would display a higher performance for photocatalytic water reduction.

## 3 Strategies for Improving MOF Photocatalytic Water‐Splitting Performance

Although MOFs materials have been considered as kinds of promising candidates for being applied in the field of photocatalytic water reduction, however, there still exist many dilemmas that have to be overcome. 1) Most MOF materials have a narrowed light absorption region, which can only be excited by UV light. 2) The bandgap of some MOFs materials is not suitable for the reaction of photocatalytic water reduction. 3) The electron–hole pairs’ separation ability needs further improvement. Therefore, it is particularly important to modify the properties of MOF materials to achieve high‐efficiency photocatalytic hydrogen production performance. In this section, the strategies for improving MOF photocatalytic hydrogen production performance are summarized based on the two main advantages of MOF materials, including tailored structure (ligand functionalization, metal doping, defect engineering, and morphology regulation). Also with their unique porosity and large BET (loading guest molecules co‐catalysts (metal, non‐metal), photosensitizer, constructing heterostructures, as templates to construct porous traditional semiconductors). The strategies for improving MOFs photocatalytic hydrogen production performance are shown in **Table** **3** and **4**.

**Table 3:** Summary of photocatalytic H_2_ production performance of MOFs decorated by different strategies based on their tailored structure

Strategies	Photocatalysts	Surface area [m^2^g^−1^]	Irradiation	Sacrificial agents	Cocatalysts	H_2_ production rate	Recycled times	Reference
Ligand functionalization	Pt/Ti‐MOF‐NH_2_	910	Visible	TEOA	Pt	3.67 μmol h^−1^	3	[70]
	MIL‐125‐(SCH_3_)_2_	1101.57	Visible	TEOA	Pt	3814 μmol g^−1^ h^−1^	–	[71]
	Pt/UiO‐66‐(SCH_3_)_2_	488	Visible	Ascorbic acid	Pt	3871 μmol g^−1^	–	[72]
	Pt/MIL‐125‐NH_2_/(OH)_2_	1147	Visible	TEA	Pt	707 μmol g^−1^ h^−1^	3	[73]
	USTC‐8(In)	1139	Visible	TEA	Pt	341.3 μmol g^−1^ h^−1^	–	[74]
	[Cd_2_(BODIPY)_2_(BPDC)_2_	–	Visible	TEA	–	60.4 mmol g^−1^ h^−1^	3	[75]
	Pt/[Zn_2_(BODIPY)(BPDC)_2_]·H_2_O	–	Visible	Ascorbic acid	Pt	4680 μmol g^−1^ h^−1^	3	[76]
	Eosin Y‐based[Cd_2_(COO)_2_(*μ*_2_‐H_2_O)]	–	Visible	TEA	–	TON = 13 920	–	[77]
Metal doping	Cu‐NH_2_‐MIL‐125(Ti)	–	Visible	TEA	Pt	490 μmol g^−1^ h^−1^	5	[78]
	Pd/CeMIL‐101	1290	Visible	–	Pd	495 μmol h^−1^	–	[79]
	Pt/NH_2_‐UiO‐66(Zr/Ti)	–	Visible	TEOA	Pt	0.38 mmol h^−1^	–	[45]
	UiO‐66(Zr/Ce/Ti)	1019	Visible	–	–	210 μmol g^−1^	–	[81]
	Pt/UiO‐66‐NH_2_	948	UV−vis	TEOA	Pt	381.2 μmol g^−1^ h^−1^	10	[90]
Defect regulation	D‐MIL‐125(Ti)–NH_2_	–	Visible	TEOA	–	10.1 μmol	3	[91]
	MET‐Cu‐D	–	Visible	TEA	–	12.91 μmol g^−1^	4	[92]
Morphology regulation	NH_2_‐MIL‐125(Ti)	1256.3	Visible	TEOA	–	60.8 μmol g^−1 ^h^−1^	3	[100]

**Table 4:** Summary of photocatalytic H_2_ production performance of MOFs decorated by different strategies based on their unique porosity and large BET

Photocatalysts	Surface area [m^2^g^−1^]	Irradiation		Sacrificial agents		Cocatalysts	H_2_ production rate	Recycled times	Reference
Pt@Zr_6_(*μ*_3_‐O)4(*μ*_3_‐OH)_4_(L_2_)6·64DMF	–	Visible		TEA		Pt	TON = 7000	4	[102]
Pt@UiO‐66‐NH_2_	867	UV−vis		TEOA		Pt	257.38 μmol g^−1^ h^−1^	5	[54]
Pt@MIL‐125/Au	986	Visible		TEOA		Au, Pt	1743 μmol g^−1^ h^−1^	–	[103]
(UCNPs)‐Pt@MOF/Au	85	UV−vis		TEOA		Au, Pt	280 μmol g^−1^ h^−1^	4	[104]
Au nanodots@thiol‐UiO‐66@ZnIn_2_S_4_	215.1	Visible		Na_2_S/Na_2_SO_3_		Au	39.2 mmol g^−1 ^h^−1^	6	[105]
g‐C_3_N_4_/UiO‐66/Ni_2_P	77.8476	Visible		TEOA		Ni_2_P	40 μmol h^−1^	4	[106]
Ni_2_P@UiO‐66‐NH_2_	726.6	Visible		TEA		Ni_2_P	409.1 μmol g^−1^ h^−1^	3	[107]
Ti_3_C_2_@MIL‐NH_2_	53.7	UV−vis		TEOA		Ti_3_C_2_	4383.1 μmol g^−1^ h^−1^	4	[111]
ErB dye sensitized Pt‐UiO‐66	–	Visible		Ascorbic acid		Pt	4.6 μmol h^−1^	3	[112]
Eosin Y (EY)‐sensitized UiO‐66‐NH_2_	550	Visible		TEOA		Pt	2760 μmol g^−1^ h^−1^	5	[113]
Calix‐3‐sensitized Pt@UiO‐66‐NH_2_	–	Visible		Methanol		Pt	1528 μmol g^−1^ h^−1^	3	[114]
UNiMOF/g‐C_3_N_4_	–	Visible		TEOA		–	20.03 μmol h^−1^	3	[121]
CD/CdS@MIL‐101	1109	Visible		Lactic acid		CD, CdS	14.66 μmol h^−1^	4	[124]
CdS/Ni‐MOF	27.8	Visible		Lactic acid		CdS	2508 μmol g^−1^ h^−1^	5	[125]
Cd_0.2_Zn_0.8_S@UiO‐66‐NH_2_	266	Visible		Na_2_S/Na_2_SO_3_		–	5846.5 μmol g^−1^ h^−1^	4	[126]
CdS/[Ni(phen)(oba)]_n_·0.5nH_2_O	–	Visible		Lactic acid		–	45 201 μmol g^−1^ h^−1^	3	[127]
ZnIn_2_S_4_@NH_2_‐MIL‐125(Ti)	521.8	Visible		Na_2_S/Na_2_SO_3_		–	2204.2 μmol g^−1^ h^−1^	5	[128]
CFB/NH_2_‐MIL‐125(Ti)	269.2	UV−vis		TEOA		Pt	1.123 mmol g^−1^ h^−1^	4	[130]
CdLa_2_S_4_/MIL‐88 A(Fe)	28.5	Visible		Ethanedioic acid		–	7677.5 μmol g^−1^ h^−1^	6	[133]
Pt‐Zn_3_P_2_‐CoP	122	UV−vis		Methanol		Pt	9.15 mmol g‐^1^ h^−1^	5	[134]
P‐ZIF‐67	–	UV−vis		TEOA		–	63.4 μmol h^−1^	–	[135]
Pd/TiO_2_	86.772	UV−vis		Methanol		Pt	979.7 μmol h^−1^	3	[136]
Bi_0.5_Y_0.5_VO_4_	–	UV−vis		–		Pt	124.2 μmol h^−1^	4	[137]
In_2_O_3_ phase junction	262	UV−vis		TEOA		–	2244 μmol g^−1^ h^−1^	5	[138]
C‐ZIF/g‐C_3_N_4_	–	Visible		TEOA		–	32.6 μmol h^−1^	4	[139]
ZnO/ZnS	–	Visible		Na_2_S/Na_2_SO_3_		–	415 μmol g^−1^ h^−1^	8	[140]
Fe−Ni−P nanotubes	–	Visible		TEOA		–	5420 μmol g^−1^ h^−1^	4	[141]

### 3.1 The Tailored Structure of MOFs

#### 3.1.1 Ligand Functionalization

**Figure 10:**
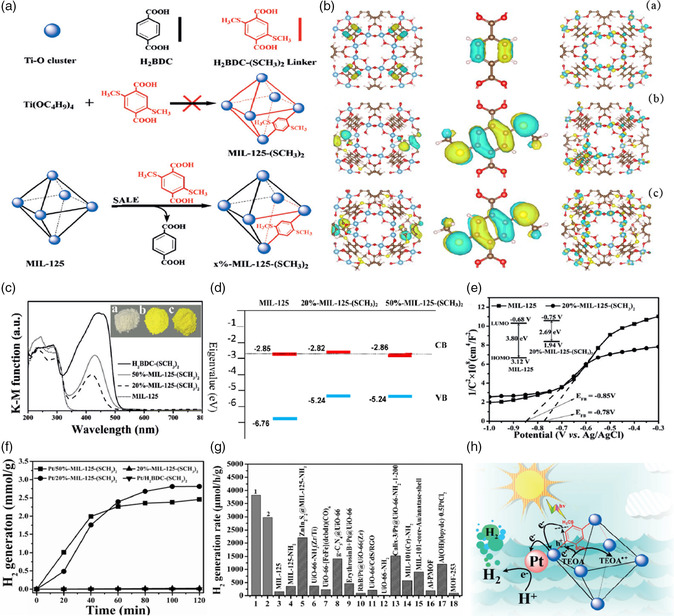
a) Schematic diagram of the SALE process to obtain *x*%‐MIL‐125‐(SCH_3_)_2_ using MIL‐125 as the parent MOF and H_2_BDC‐(SCH_3_)_2_ as the exterior exchange linker. The cage represents the cavity in the MOF structure. b) Frontier orbitals of (a) MIL‐125, (b) 20%‐MIL‐125‐(SCH_3_)_2_, and (c) 50%‐MIL‐125‐(SCH_3_)_2_. The orbitals at the left (right) are for the VB (CB), and those in the middle are enlarged figures near the benzene rings for the VB. c) UV/vis spectra. Inset: powder colors of a) MIL‐125, b) 20%‐MIL‐125‐(SCH_3_)_2_, and c) 50%‐MIL‐125‐(SCH_3_)_2_. d) The calculated VB and CB energies of MIL‐125 and *x*%‐MIL‐125‐(SCH_3_)_2_. e) Mott−Schottky plots of MIL‐125% and 20%‐MIL‐125‐(SCH_3_)_2_ in 0.1  M Na_2_SO_4_. Inset in (e) shows the bandgaps of MIL‐125% and 20%‐MIL‐125‐(SCH_3_)_2_. f) Time course of photocatalytic H_2_ production under 400–800 nm. g) Activity of splitting water into H_2_ under visible light irradiation using 20 %‐MIL‐125‐(SCH_3_)_2_ (denoted as 1), 50 %‐MIL‐125‐(SCH_3_)_2_ (denoted as 2), and those representative photocatalytic MOFs. h) Photocatalytic H_2_ production mechanism of Pt/*x*%‐MIL‐125‐(SCH_3_)_2_. The blue balls represent Ti‐oxo clusters in MOF *x*%‐MIL‐125‐(SCH_3_)_2_ lattice. a–h) Reproduced with permission.^[^ ^71^ ^]^ Copyright 2018, Wiley‐VCH.

The selective introduction of organic functional groups into the framework of MOF materials can effectively reduce their optical bandgap and broaden the visible light response range. Therefore, ligand functionalization methods have been widely used to improve the photocatalytic activity of the MOF materials for water reduction. Simple amination of the target MOF‐based catalysts is one of the most common ligand functionalization strategies to improve their activity for photocatalytic H_2_ evolution. For example, Garcia and coworkers demonstrated that amino‐functionalized UiO‐66‐NH_2_, an MOF constructed by the Zr_6_ clusters and 2‐amino‐terephthalate ligands, can produce a large spectral red shift compared with UiO‐66 (containing terephthalate ligand without functional groups).^[^ ^44^ ^]^ The bandgap of MOF photocatalysts can be significantly reduced by decorating the amino groups in their framework, the photoresponse range can be expanded from UV light to visible light and even UV light, and then the recombination of photogenerated electrons and holes can also be reduced. Therefore, the photocatalytic water decomposition performance of the amino‐modified UiO‐66 can be enhanced. Inspired by earlier work, an amino‐functionalized Ti‐MOFs, MIL‐125‐NH_2_, was also studied in the photocatalytic water reduction by Masaya Matsuoka et al. through introducing 2‐aminophthalic acid into the framework of Ti‐MOFs. Their light response range was expanded from UV light to visible light, and the highly efficient photocatalytic hydrogen production could be conducted in the aqueous solution with TEOA as sacrificial agent.^[^ ^70^ ^]^ Based on earlier works, researchers gradually recognized that ligand functionalization is a favorable method for decorating the MOFs‐based photocatalysts and extending their light absorption region. Therefore, different functional groups, including −Br, −OH, −SH, and −NO_2_, have been selected to improve the light absorption region of the MOF materials. However, their efficiency is lower than that of −NH_2_ groups. It is commendable that Guo and coworkers successfully constructed a highly photoactive visible light‐driven material by decorating MIL‐125 with methylthio (SCH_3_)_2_ groups via a solvent‐assisted ligand‐exchange (SALE) method.^[^ ^71^ ^]^ By decorating the ligands with methylthio groups, the light absorption region of MIL‐125 can be extended from 345 to 525 nm, thus responding to visible light, which was wider than that of −NH_2_‐decorated MIL‐125. Correspondingly, the bandgap can be decreased from 3.6 to 2.6 eV. Consequently, using Pt as cocatalyst and triethanolamine as sacrificial agent, the methylthio‐functionalized MOFs exhibited excellent performance for H_2_ production with a high quantum yield of 8.90% at 420 nm, which was higher than most reported Pt/MOF‐based photocatalysts (**Figure** **10**). The electron‐donating ability, dimensions, and geometry of the functional groups played an important role in determining the band structure of the MOF‐based photocatalysts.

In this regard, Li's group successfully constructed three isostructural UiO‐66‐based MOFs decorated with three different sulfur‐containing groups (X = −SCH_3_, −SH, −SO_3_H), which possessed different electronic natures, molecule dimensions, and geometry.^[^ ^72^ ^]^

**Figure 11:**
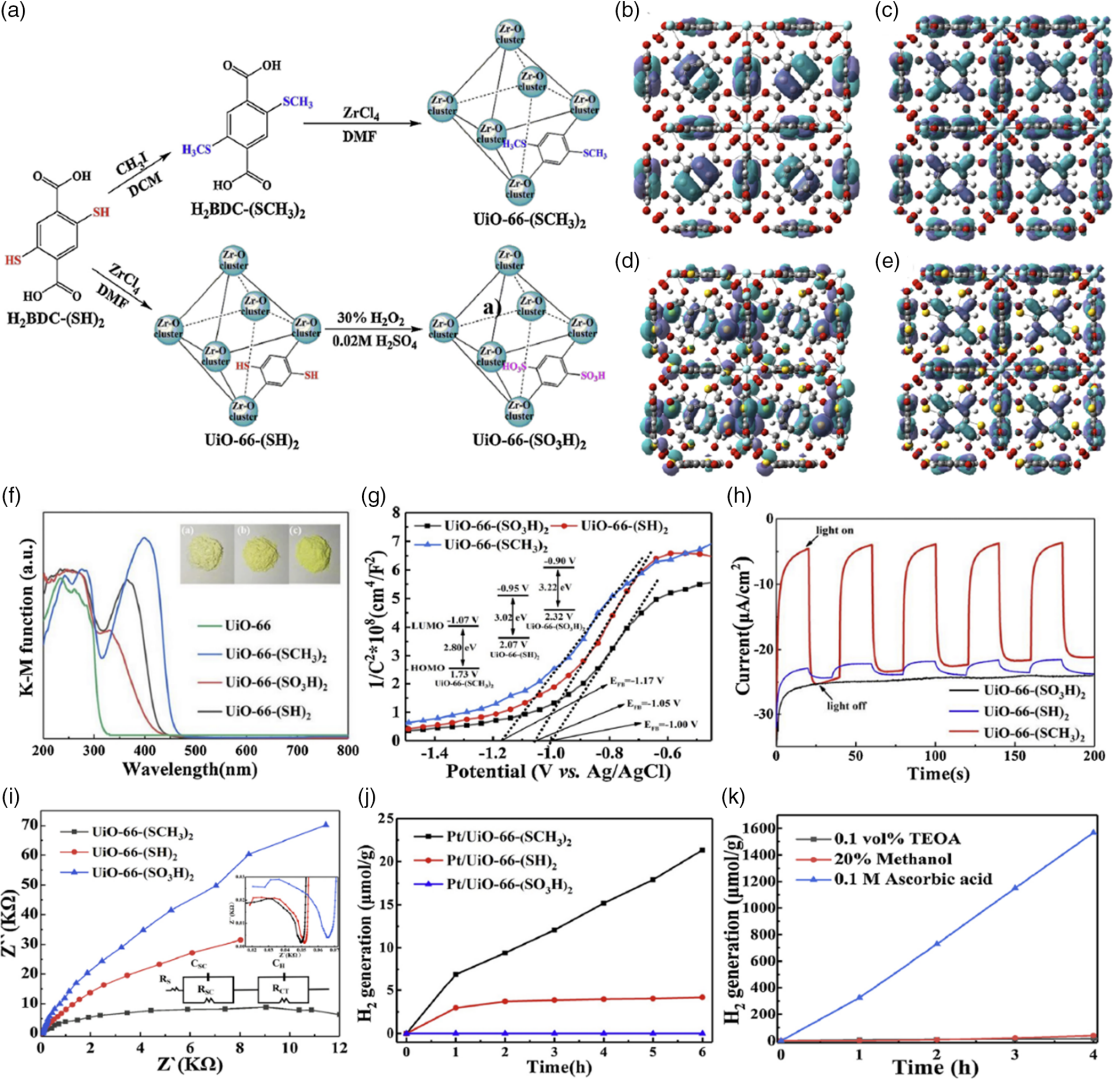
a) Synthesis diagram of isostructural UiO‐66‐(SH)_2_, UiO‐66‐(SCH_3_)_2_, and UiO‐66‐(SO_3_H)_2_ possessing the parent structure of UiO‐66. The octahedral cage represents the pore structure within UiO‐66‐X_2_. b) highest occupied crystal orbital (HOCO) and c) lowest unoccupied crystal orbital (LUCO) of UiO‐66 and d) HOCO and e) LUCO of UiO‐66‐(SH)_2_. f) UV–vis absorption spectra of UiO‐66‐X_2_ in this study. g) Current/time plots of UiO‐66‐X_2_ measured at an applied bias potential of 0.19 V versus RHE with and without visible light irradiation (> 400 nm) pulse of 20 s. h) Nyquist and i) Mott−Schottky plots of UiO‐66‐X_2_ in 0.1 M H_2_SO_4_. j) Time course of H_2_ production of Pt/UiO‐66‐X_2_ from aqueous solution with 0.1 vol% TEOA as electronic sacrificial reagent at room temperature. k) Time course of H_2_ production of Pt/UiO‐66‐(SCH_3_)_2_ with 0.1 M ascorbic acid, 20 vol% methanol, and 0.1 vol% TEOA as sacrificial electron donors in comparison. a–k) Reproduced with permission.^[^ ^72^ ^]^ Copyright 2019, Elsevier.

Then, the influence of the functional groups for determining their bandgap and photoactive properties was systematically studied. Due to the strong electron‐donating ability and disubstituted function group effect, the optical absorption edge belonging to −SCH_3_ displayed the largest red shift, and ‐SCH_3_ groups proved to be the most effective group for improving the light absorption ability among three isostructural MOFs. Finally, excellent photocatalytic hydrogen production activity was obtained (**Figure** **11**). Very recently, the photocatalytic hydrogen production performance of amino (−NH_2_) and/or hydroxyl (−OH) MIL‐125 was also investigated by Stylianou and coworkers.^[^ ^73^ ^]^ Three different functionalized ligands (NH_2_)_2_‐BDC, OH‐BDC, and (OH)_2_‐BDC, were embedded into the framework of MIL‐125 to form a *π*‐conjugated structure consisting of weakly coupled single‐chromophore functionalized units.

The UV spectrum showed that MIL‐125‐NH_2_/(OH)_2_ had the largest molar extinction coefficient, and the visible light absorption range was broadened. The enhanced P–π conjugation resulted in accelerated carrier separation and extended carrier lifetime and finally achieved an enhanced H_2_ production rate up to 707 μmol h^−1 ^g^−1^.

In addition to the aforementioned functional groups, porphyrin units, which have been demonstrated to possess excellent visible light‐harvesting capability, have also been widely used as ligands to synthesize MOF materials with visible light absorption ability. Moreover, there also exist four unsaturated coordinated N atoms at the center of the porphyrin ring, which can serve as a coordinated center to load metal ions, thus promoting the charge carrier separation and enhancing the photocatalytic activity. For example, Jiang and coworkers first reported the preparation of a highly stable out‐of‐plane (OOP) porphyrin MOF USTC‐8 (In) using In(OH)_3_ as precursor.^[^ ^74^ ^]^ In this material, the In ions not only exist in the metal chains, but also coordinate in the center of the porphyrin ring. Benefitting from the wide light absorption region of the porphyrin units, In‐MOF displayed excellent ability for visible light absorption. Moreover, the presence of indium atoms located above the porphyrin plane can also undergo a detachment–insertion process during the photocatalytic reaction, which can greatly improve the electron–hole separation ability. Thanks to this, it showed an excellent photocatalytic activity for water reduction, in which the H_2_ evolution rate can reach up to 341.3 μmol g^−1 ^h^−1^.

**Figure 12:**
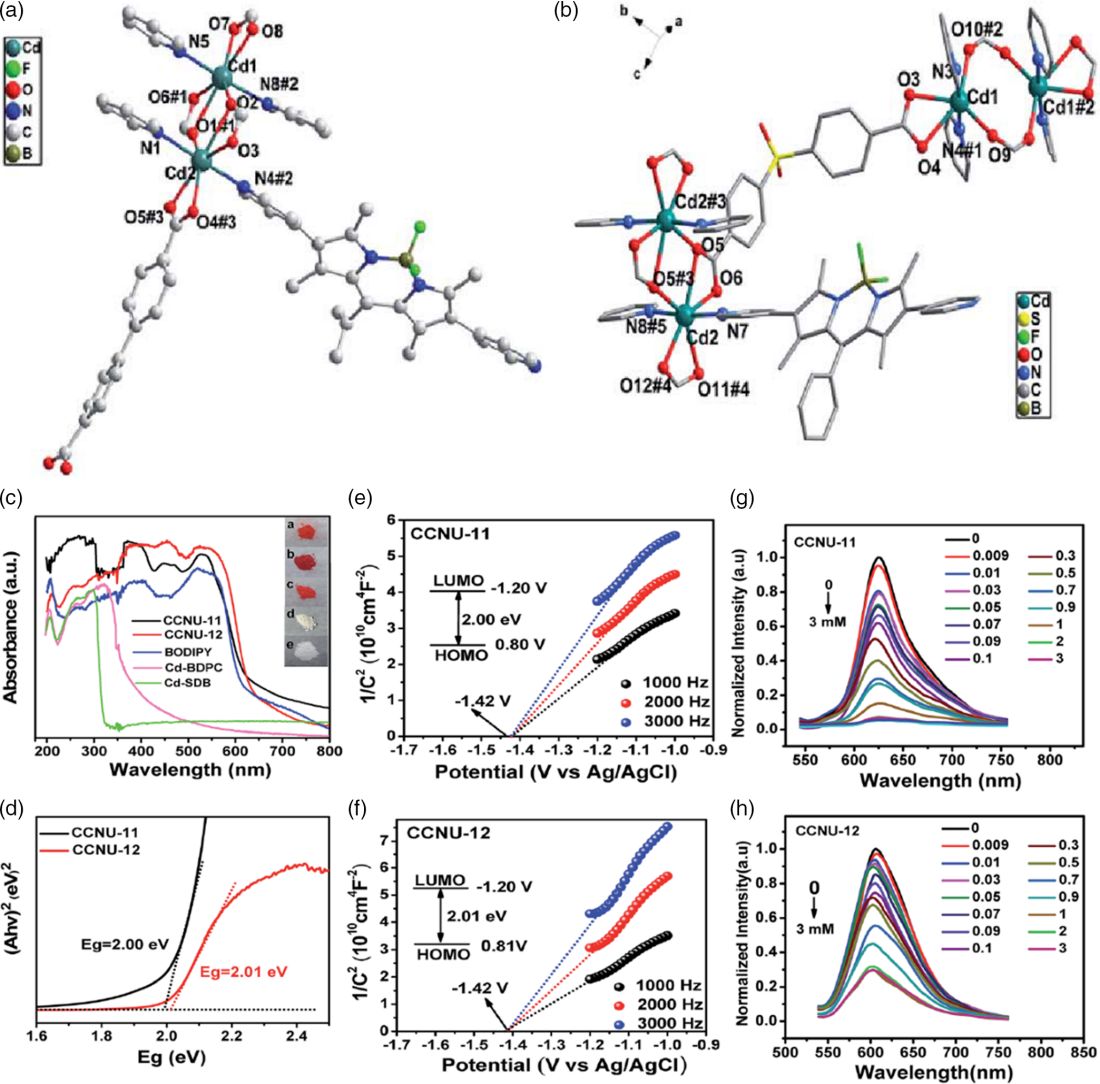
CCNU‐11: a) coordination environment of Cd^II^; CCNU‐12: b) coordination environment of Cd^II^. c) Solid UV/vis spectra of CCNU‐11, CCNU‐12, BODIPY, Cd‐BPDC, and Cd‐SDB (inset shows the images (a), (b), (c), (d), and (e) corresponding to CCNU‐11, CCNU‐12, BODIPY, Cd‐BPDC, and Cd‐SDB). d) Tauc plots of CCNU‐11 and CCNU‐12. Mott–Schottky plots for e) CCNU‐11 and f) CCNU‐12 f) in a 0.4 M Na_2_SO_4_ aqueous solution. (Inset) Energy diagram of the highest occupied molecular orbital (HOMO) and lowest unoccupied molecular orbital (LUMO) levels. The emission spectra of g) CCNU‐11 and h) CCNU‐12 h) in water suspension upon the addition of [Co(bpy)_3_]Cl_2_ (*E*_*x*_ = 440 nm). The final concentration of [Co(bpy)_3_]Cl_2_ is indicated in the mM unit. a–h) Reproduced with permission.^[^ ^75^ ^]^ Copyright 2019, The Royal Society of Chemistry.

Apart from the aforementioned examples, photosensitizer units can also be applied to construct MOF‐based photocatalysts with visible light absorption ability. For example, pyridine‐functionalized boron dipyrromethene (BODIPY) units possess large electron delocalization systems and have excellent response for visible light. Inspired by this, Wen et al. synthesized two 3D MOFs, named, CCNU‐11 and CCNU‐12, through a mix‐ligand method, in which 4,40‐biphenyldicarboxylic acid (H_2_BPDC) or 4,40‐sulfonyldibenzoic acid (H_2_SDB) was used to form the layer structure, and the BODIPY‐based ligand was applied as the pillar to form the 3D structure.^[^ ^75^ ^]^ In virtues of the excellent light‐harvesting ability, both two materials displayed high performance for photocatalytic H_2_ evolution. During the photocatalytic reaction, the BODIPY photosensitizer first excited electrons under visible light irradiation, then were injected into the [Cd_2_(COO)_2_] cluster, and finally transferred to [Co(bpy)_3_]Cl_2_, which resulted in a slower photogenerated carrier current subcombination rate. Finally, the water was efficiently reduced to hydrogen on the surface of the catalyst (**Figure** **12**). Similar to earlier work, they also used this BODIPY ligand to successfully construct novel Zn‐based MOFs, which also exhibited excellent activity for photocatalytic H_2_ production.^[^ ^76^ ^]^ Apart from BODIPY ligand, other photosensitizers, such as Eosin Y‐based ligands, can also be introduced into MOFs as organic linkers to improve the visible light absorption range of MOF‐based materials. For instance, through combining [Cd_2_(COO)_2_(*μ*_2_‐H_2_O)] secondary building units and Eosin Y‐based ligands, a porous Cd‐based MOF was successfully constructed by Wu's group.^[^ ^77^ ^]^ Due to excellent photoactivity of the Eosin Y‐based ligands, such MOFs displayed high photocatalytic activity for water reduction even under visible light irradiation. Based on earlier works, we can recognize that ligand functionalization method is a versatile and reliable tool for improving the photocatalytic activity for H_2_ evolution of the MOF‐based photocatalysts.

#### 3.1.2 Metal Doping

In addition to ligand functionalization method, metal doping strategy has also been considered as an effective tool for optimizing the photocatalytic performance of MOF‐based photocatalysts. There are two ways for conducting the metal doping strategy in terms of MOF materials. One is that the doping metal ions can coordinate with the organic ligand containing functional groups with lone pair of electrons, such as amino, sulfhydryl, and porphyrin units. The doped metal can act as a medium to promote electron transfer and greatly improve the photocatalytic activity. One of the most fascinating examples was reported by Lu's group.^[^ ^78^ ^]^ In their work, NH_2_‐MIL‐125(Ti), which was constructed by TiO_*x*_ clusters and NH_2_‐containing BDC linkers, was decorated with the highly active Cu^II^/Cu^I^ as mixed center through a simple doping method. During the photocatalytic process, the organic ligand can generate electron–hole pairs under visible light irradiation. Due to the presence of Cu atoms coordinated with the amino groups, the electrons were prone to be transferred from −NH_2_ to Cu^II^ and, then Cu^II^ was partially reduced to form active Cu centers with mixed valence, instead of original TiO_*x*_ nanoclusters. Thanks to this, the length of the electron transfer pathway was greatly shortened. Thus, the lifetime of photogenerated carriers and carrier density were greatly increased 27‐fold and 7000‐fold, respectively, which can be indicated by the CV, current–time plots, Nyquist plots, and femtosecond transient absorption (TA) spectra.

**Figure 13:**
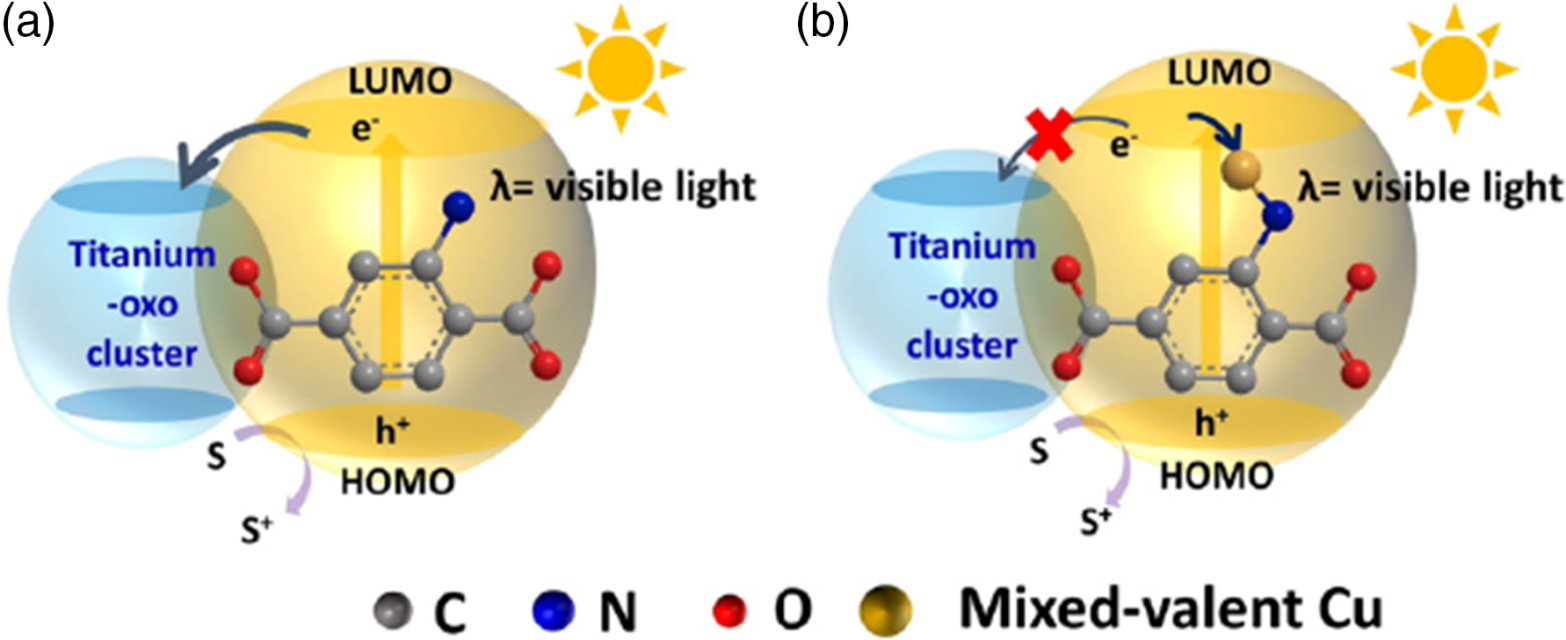
A schematic illustration showing the electron‐transfer pathways from an organic linker to a) a titanium‐oxo cluster in NH_2_‐MIL‐125(Ti) and to b) a mixed‐valence Cu center. S represents a sacrificial agent. Reproduced with permission.^[^ ^78^ ^]^ Copyright 2020, Wiley‐VCH.

In comparison with previous work, which usually applied Pt atoms as cocatalysts, this work provides a unique method for designing photocatalysts with high activity for photocatalytic H_2_ production through doping non‐noble mixed‐valence Cu atoms as cocatalysts (**Figure** **13**).

**Figure 14:**
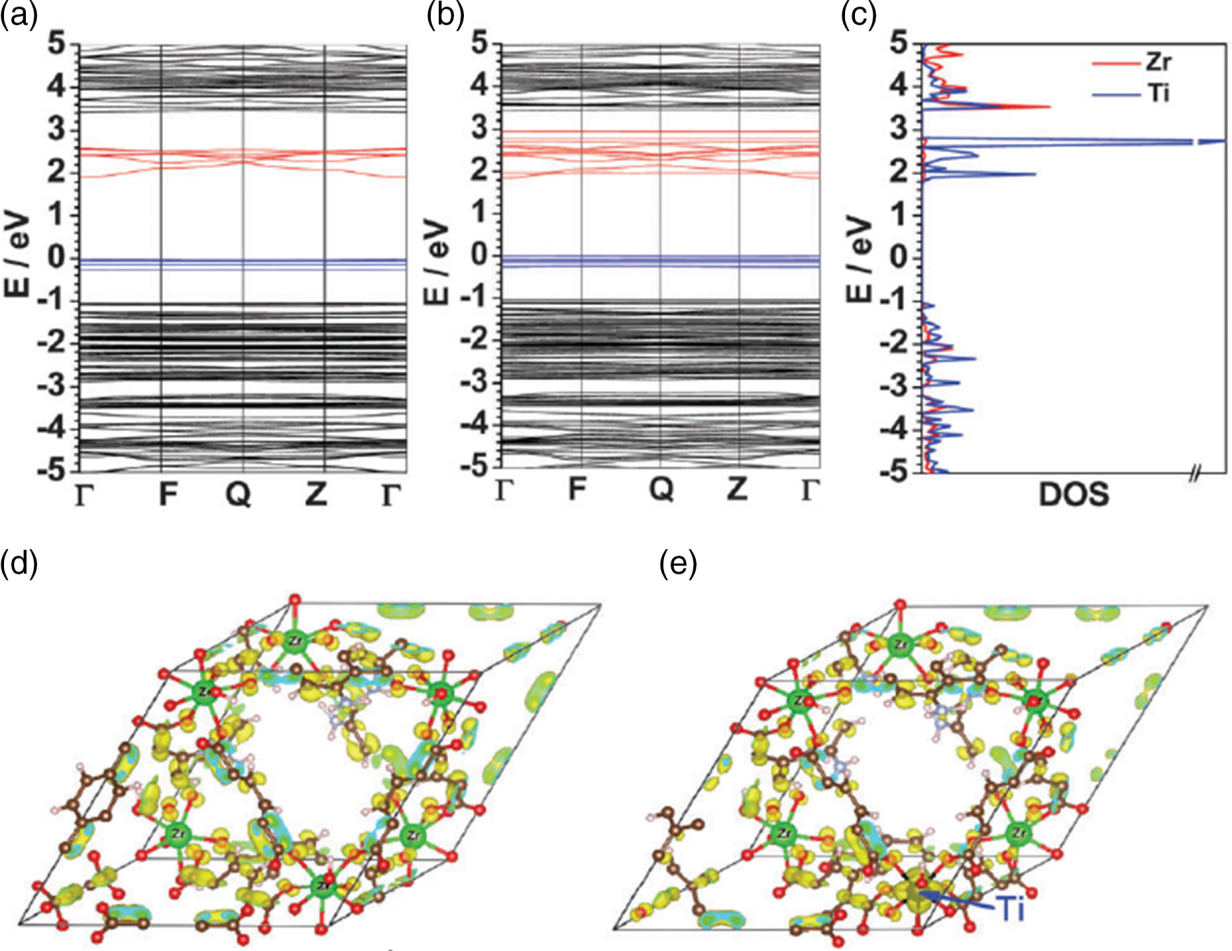
a) Band structure of NH_2_‐Uio‐66(Zr); b) band structure of Ti‐doped NH_2_‐Uio‐66(Zr); c) partial density of states (DOSs) of Zr and Ti atoms in the Ti‐doped NH_2_‐Uio‐66(Zr) and the partial charge density map (drawn at the isosurface level: 0.01 e Å^−3^) of the CB located in the region from 1.9 to 2.6 eV above the Fermi level for the d) undoped and e) Ti‐doped NH_2_‐Uio‐66(Zr). In (a–c), the Fermi level is set to zero. a–e) Reproduced with permission.^[^ ^45^ ^]^ Copyright 2015, The Royal Society of Chemistry.

In addition to doping ions at the ligand modification site, the other way is to decorate the metal clusters with other metals. For example, doping Ti ions, which displayed a higher redox activity, into the Zr_6_ cluster of MOF structure can significantly improve their photocatalytic activity for H_2_ evolution. Li and coworkers prepared the Ti‐substituted NH_2_‐UiO‐66(Zr/Ti) using a postsynthetic exchange (PSE) method.^[^ ^45^ ^]^ The density functional theory (DFT) calculations verified that substituted Ti can act as an electron mediator to improve the photocatalytic H_2_ production performance (**Figure** **14**). Moreover, the Ti‐mediated electron transfer revealed that Ti^3+^ donates an electron to Zr^4+^ to promote the formation of Zr^3+^.

The substituted Ti center in NH_2_‐UiO‐66(Zr/Ti) accelerated the separation of carriers from excited ATA to Zr‐O oxo‐clusters, thus achieving enhanced photocatalytic performance. Under the same conditions, the hydrogen production rate of Pt/NH_2_‐UiO‐66 (Zr/Ti) was 1.5 times higher than that of Pt/NH_2_‐UiO‐66 (Zr). Another fascinating example was reported by Yamashita and coworkers. They doped Ce ions into the nodes of amine‐functionalized MIL‐101 and obtained a new hybrid MOF named Ce‐MIL‐101.^[^ ^79^ ^]^ Attributed to the hypochromic effect, the intensity of the spectral absorption band was significantly reduced after doping the MIL‐101 with Ce ions. At the same time, due to the (Ce^4+^/Ce^3+^) redox cycle, the photogenerated carrier separation ability was enhanced, and finally exhibited an excellent photocatalytic activity, in which the hydrogen production of Pd/CeMlL‐101 was 2.3 times higher than that of Pd/MlL‐101 and can reach up to 495 μmol h^−1^.

In addition to doping one kind of metal ion, doping multiple metal ions can also affect the electron transfer in MOFs materials, thus promoting water reaction. For example, Musho and coworkers used three inorganic metal ions (Zr, Ti, Hf) and three functional groups including amino group (NH_2_), nitro group (NO_2_), and a hydrogenated case (H) for constructing functionalized UiO‐66‐series MOFs and studied its effect on the optical bandgap of catalytic materials.^[^ ^80^ ^]^ Through theoretical research, the results can be concluded that when Ti is completely replaced, the smallest predicted bandgap of UiO‐66‐series MOFs can even reach 1.62 eV. This research theoretically explains that the metal cluster substitution with metal ions can change the optical properties of MOF‐based catalytic materials, thus in turn affecting their photocatalytic activity. Recently, García's group successfully designed a series of UiO‐66 MOFs with mixed metal clusters (M: Zr, Zr/Ti, Zr/Ce, Zr/Ce/Ti, Ce).^[^ ^81^ ^]^ Among them, the trimetallic MOFs UiO‐66, in which Ti shared nodes both with Zr and with Ce in UiO‐66 (Zr/Ce/Ti), as revealed by the X‐ray photoelectron spectroscopy (XPS) result, displayed the highest photocatalytic activity. As revealed by a series of spectroscopy and photoelectric tests, the modification of node composition can significantly adjust the light absorption capacity of the catalytic materials and support faster photogeneration carrier separation, subsequently increasing the photocatalytic activity of the catalytic material. It is worth mentioning that such photocatalysts can achieve overall water splitting, in which the H_2_ and O_2_ evolution rate can reach up to 230 and 110 μmol g^−1^ under UV light and 210 and 70 μmol g^−1^ under visible light, respectively.

#### 3.1.3 Defect Regulation

**Figure 15:**
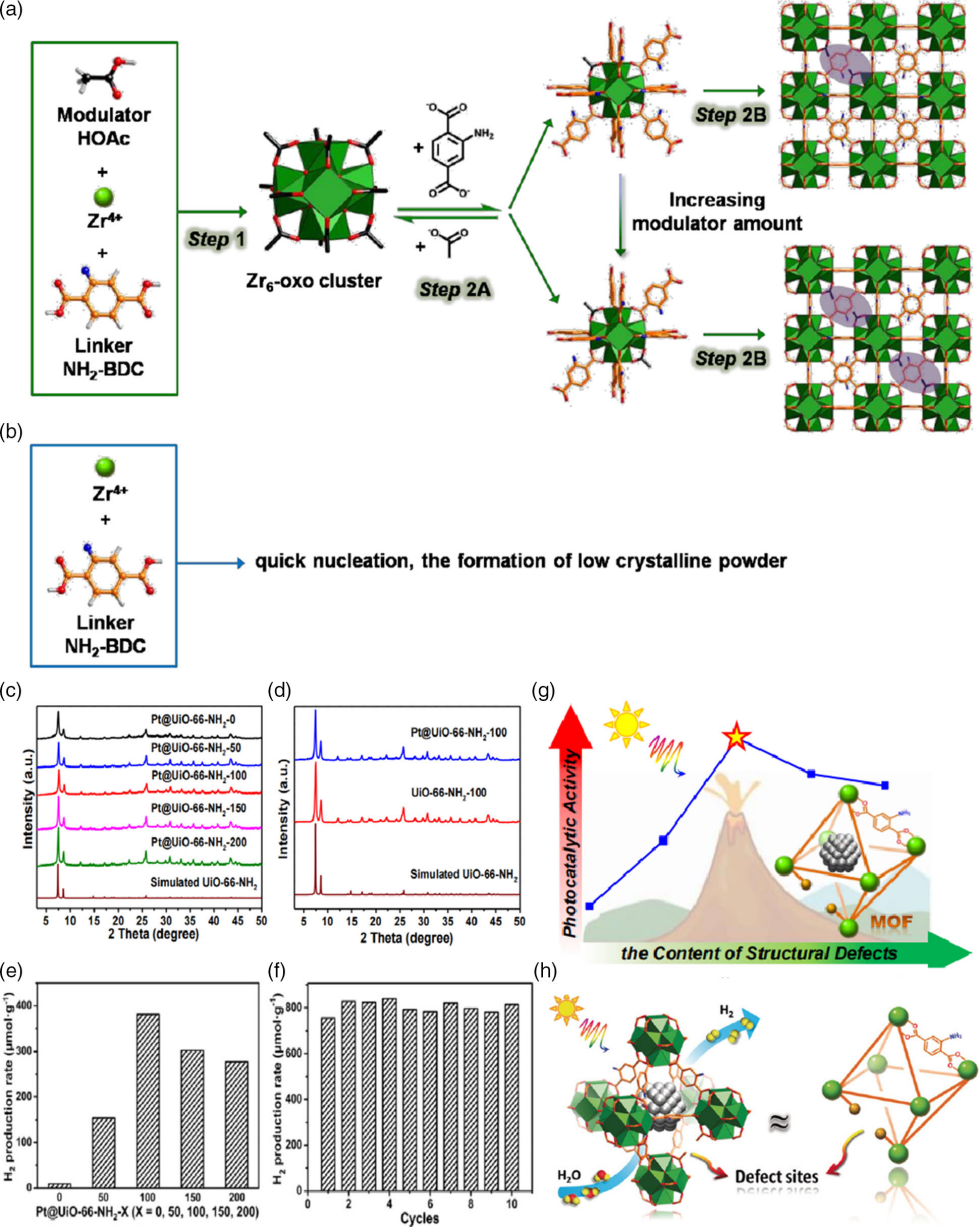
Illustration showing a) the proposed formation process of defective UiO‐66‐NH_2−*X*_ via a modulator approach and b) the synthesis of UiO‐66‐NH_2_ without modulator. The purple ellipses schematically indicate the defects in the MOF. c) Powder XRD patterns of Pt@UiO‐66‐NH_2−*X*_ (*X* = 0, 50, 100, 150, 200) and d) particular comparison of powder XRD profiles for UiO‐66‐NH_2_‐100 and Pt@UiO‐66‐NH_2_‐100. e) Photocatalytic H_2_ performance from water splitting over different catalysts in MeCN/TEOA/H_2_O (10.8:1:0.2 v/v, 30 mL) under light irradiation. f) Recycling performance of Pt@UiO‐66‐NH_2_‐100 (2 h per cycle). g) Schematic illustration showing the relationship between the content of structural defects and the photocatalytic activity. h) Photocatalytic hydrogen production over Pt@UiO‐66‐NH_2_‐*X* with structural defects. a–h) Reproduced with permission.^[^ ^90^ ^]^ Copyright 2019, Wiley‐VCH.

**Figure 16:**
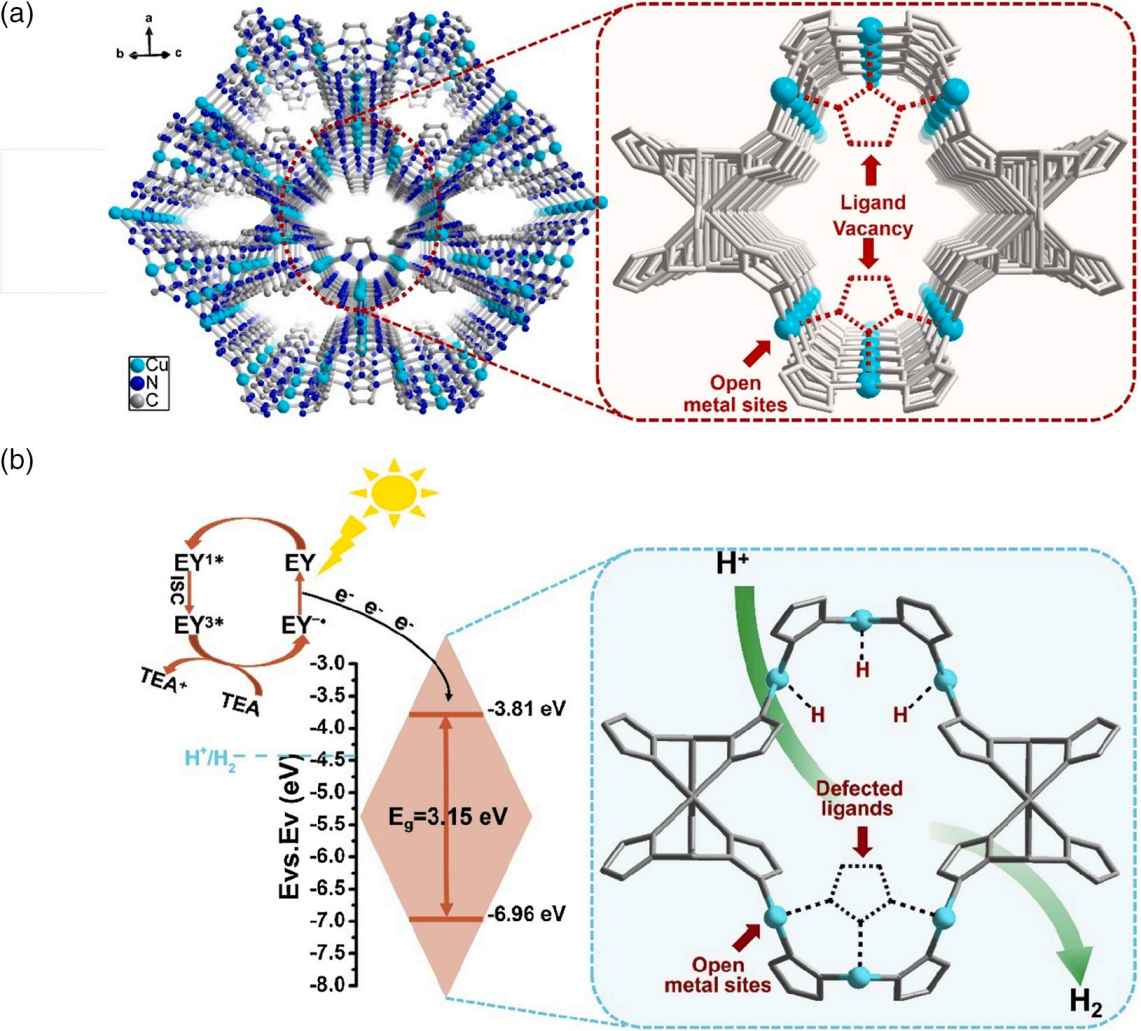
a) Schematic illustration showing MET‐Cu and structural defects in MET‐Cu‐D. b) Photocatalytic H_2_ production mechanism of MET‐Cu‐D. a,b) Reproduced with permission.^[^ ^92^ ^]^ Copyright 2021, Elsevier.

In previous reports, defecting engineering methods have usually been used to modify the inorganic semiconductor materials properties, including electronic and band structures, surface reactive sites, conductivity, and charge carrier concentration, thus optimizing their photocatalytic activity for water reduction.^[^ 82, 83, 84, 85, 86, 87, 88, 89 ^]^ However, structure defects for influencing the photocatalytic performance of MOF‐based materials have rarely been reported. In comparison with traditional inorganic semiconductor materials, the periodicity of MOF materials and the well‐defined structures are good places to build defects. Therefore, in recent years, the introduction and control of defects to improve the photocatalytic activity of MOF‐based photocatalysts have attracted tremendous interests and became one of the hottest research spots in the field of photocatalytic water splitting. The first example for exploring the effect of MOF defects for determining the photocatalytic water reduction performance was reported by Jiang's group.^[^ ^90^ ^]^ In their work, UiO‐66‐NH_2_ material was used as a platform to build controllable concentration in their framework by tuning the addition of water and acetic acid (HOAc) modulator. Then studied the relationship between structural defects and the performance of photocatalytic hydrogen production in detail (**Figure** **15**). Interestingly, it showed a volcano‐type trend, and there was an optimal value for the concentration of defects. At first, the increase in defect concentration was accompanied by an increase in active sites, but when the defect concentration was too high, these active sites became new recombination regions for photogenerated electrons and holes, which were not conducive to photocatalytic activity. Based on earlier work, we can recognize that like other inorganic semiconductor materials, the defect engineering method can also be used in the field of MOF materials, effectively regulating their photocatalytic performance. However, it is particularly important to construct a reasonable concentration of defects in their framework. Immediately afterward, Matsuoka and his coworkers used photothermo chemical treatment to construct ligand defects on the amino‐functionalized MIL‐125(Ti).^[^ ^91^ ^]^ After visible light irradiation, the formation of Ti^3+^ can weaken the chemical bond between the ligand and Ti clusters, and then some organic ligands were eliminated after heating, thus forming new reactive sites on Ti‐oxo clusters, but still maintained the original framework. Like Jiang's reported work, MIL‐125(Ti) after defect engineering displayed ultrahigh visible light hydrogen production activity compared with the original MOF material. Subsequently, Zang's group used a solvothermal reaction to construct Cu‐MOFs with ligand vacancies in the unsaturated Cu coordination sites.^[^ ^92^ ^]^ Then, through a series of control experiments and characterizations, they first verified the existence of the vacancies and then confirmed that the existence of defects has the advantages of accelerating the transfer efficiency between photogenerated carriers, which was the main reason for achieving high‐efficiency photocatalytic reduction activity (**Figure** **16**).

#### 3.1.4 Morphology Regulation

**Figure 17:**
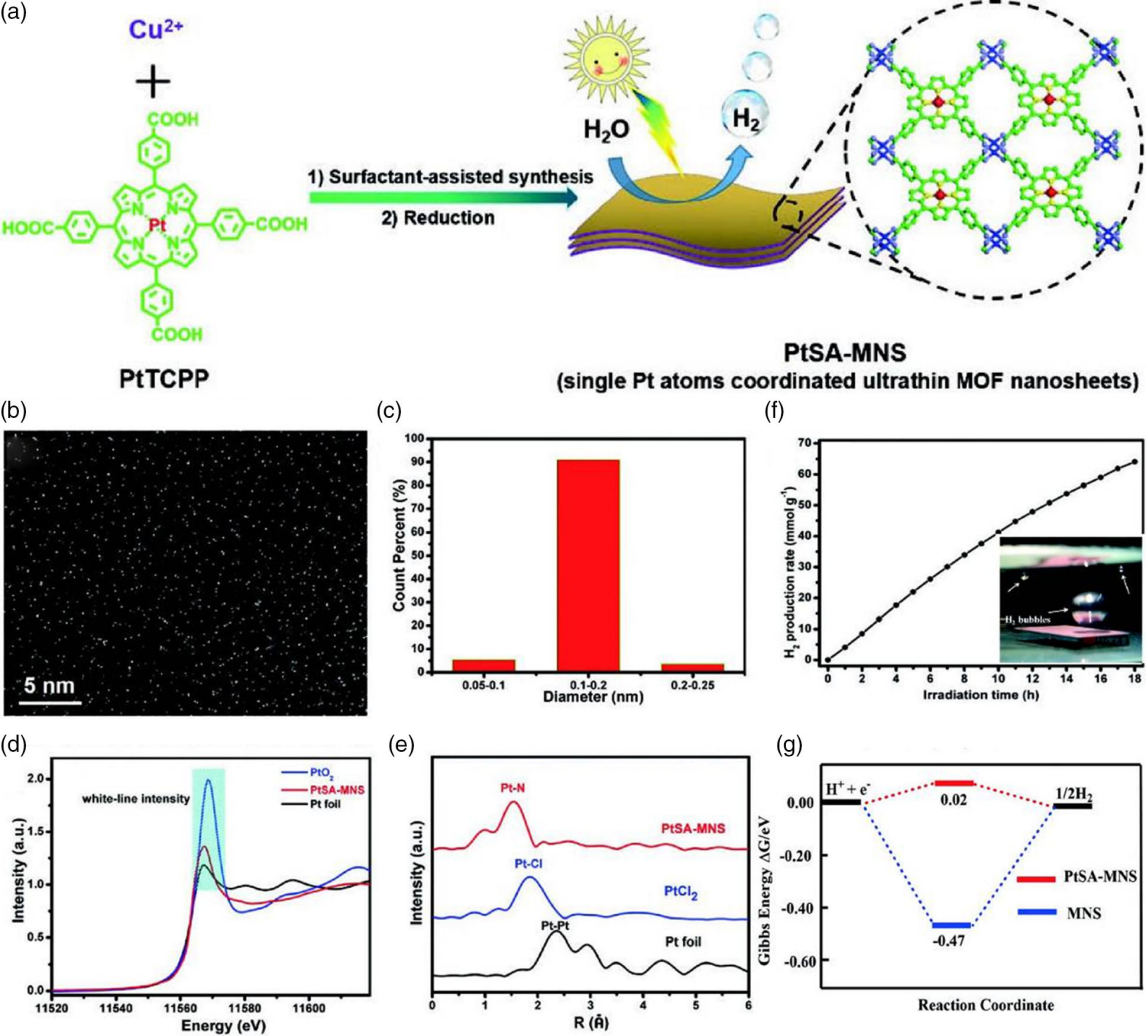
a) Illustration of the synthetic route toward Pt single‐atom‐coordinated ultrathin‐layered MOF (PtSA‐MNSs) through a surfactant‐stabilized coordination strategy for photocatalytic hydrogen production. b) Aberration‐corrected high angle annular dark field (HAADF)−Scanning transmission electron microscopy (STEM) image of the PtSA‐MNSs. c) Statistical size distribution of 200 bright spots in (b). d) The normalized Pt L3‐edge XANES spectra for PtSA‐MNSs, PtO_2_, and Pt foil. e) Pt L3‐edge EXAFS spectra for PtSA‐MNSs, PtCl_2_, and Pt foil. f) Photocatalytic H_2_ generation rate of the thin‐layered MOF on glass. g) ΔGH* on MNSs and PtSA‐MNSs, respectively. a–g) Reproduced with permission^[^ ^38^ ^]^. Copyright 2019, Wiley‐VCH.

In addition to the aforementioned methods, morphology regulation, including the crystal size, thickness, and facet regulation, has also been considered as one of the most efficient methods for regulating the photocatalytic activity of the catalysts.^[^ 93, 94, 95, 96, 97, 98, 99 ^]^ Although various MOFs with different morphologies have been reported in the literature for photocatalytic hydrogen production reaction, the influence of morphology on their activity and the relationship between the two factors have rarely been studied systematically. However, in any case, it is particularly important to deeply understand it. In comparison with the bulk MOF materials, 2D MOFs with ultrathin thickness have many advantages. 1) The reduced thickness of 2D MOFs can shorten the distance of charge carrier migration; 2) 2D MOFs possess abundant surface reactive sites; 3) the bandgap of 2D MOFs can be tuned by regulating their thickness, thus endowing them with a higher light‐harvesting ability; and 4) their ultrahigh surface area is not only beneficial for substrates or products diffusion but can also provide an ideal platform for combining other cocatalysts. Therefore, endowing MOFs with ultrathin thickness is an effective method for enhancing their photocatalytic activity for H_2_ evolution. For example, Zhou and coworkers synthesized novel 2D ultrathin MOF nanosheets coordinated with an ultrahigh concentration of monoatomic Pt using a surfactant‐stabilized coordination strategy.^[^ ^38^ ^]^ The atomic force microscope confirmed that the thickness of the nanosheets was only 2.4 ± 0.9 nm, and the specific surface area was determined to reach 570 m^2^ g^−1^ by BET analysis. Compared with the bulk material, the ultrathin MOFs displayed a higher photocatalytic activity for water reduction. The excellent results can be ascribed to the ultrathin thickness of the MOFs, which can not only disperse the monoatomic Pt with high concentration, but also effectively shorten the charge carrier migration distance, as revealed by the photoelectric test results. Consequently, under visible light irradiation, it exhibited a record‐breaking hydrogen production activity compared with previously reported MOF photocatalysts (**Figure** **17**). In addition to controlling the thickness of the MOF materials, crystal plane control is also an important strategy to improve the MOF‐based photocatalytic activity. Different facets of photocatalysts have different atomic arrangements, thus displaying distinct photocatalytic activity. In previous work, the facet‐regulating methods for optimizing the photocatalytic activity of the metal and metal oxide have been widely used. However, such a concept has been rarely studied in MOFs. In recent years, Sun and coworkers made a breakthrough in this area. Through changing the addition amount of CTAB, they obtained a series of NH_2_‐MIL‐125(Ti) with different dominated crystal faces, including {110}, {001}, {100}, and {111} facets.^[^ ^100^ ^]^ Among them, NH_2_‐MIL‐125(Ti) with the dominant {110} facet displayed the highest photocatalytic activity for hydrogen production, and the apparent quantum yield (AQY) was as high as 3.60% at 420 nm. To better understand the mechanism behind the observed activity trend, the surface structure of NH_2_‐MIL‐125(Ti) was well studied by the DFT calculations method. The reasons can be summarized that the surface energy of {110} in NH_2_‐MIL‐125(Ti) is higher than other facets, and the facet of NH_2_‐MIL‐125(Ti) possessed abundant exposed metal clusters, which can be regarded as reactive sites, thus promoting the photocatalytic activity. The earlier results can also be confirmed by a series of photoelectric tests. Although no works have been reported wherein the photocatalytic activity of MOFs for water reduction can be optimized by tuning their exposed active facets, the earlier work provides researchers with enough imagination for enhancing their activity through such methods. Tuning the particle size of the photocatalysts has been considered as a versatile tool for regulating the photocatalytic activity of the noble metals.

However, it has rarely been studied in MOFs materials. Recently, Sun's group constructed a series of nanoscaled MIL‐101(Cr) with different crystal sizes ranging from 80 to 800 nm by tuning the concentration of reaction solutions.^[^ ^101^ ^]^ Due to the high density of unit cells on corners and edges, MIL‐101(Cr) with the smallest size (80 nm) displayed the most efficient electron transfer performance, thus exhibiting high performance for CO_2_ reduction. This is the first and unique attempt to investigate the crystal size for influencing the photocatalytic activity of MOF materials for CO_2_ reduction. Similar to the facet‐regulating method, there have been no examples to investigate the crystal size of MOFs for influencing their photocatalytic activity for H_2_ production, which has great values for being investigated. Based on earlier examples, we can conclude that the main idea of the above strategies is based on the tunable structures of MOF materials. Among different strategies, the ligand functionalization and metal doping methods have been widely used in MOF materials and have great effect for determining their photocatalytic activity for water splitting. Although, the defect regulation and morphology regulation methods have rarely been studied for MOFs in the field of photocatalytic H_2_ production, they have great values for being investigated in the future.

In Table 3, we show the examples for optimizing the photocatalytic activity of MOFs for H_2_ production through ligand functionalization, metal doping, and defect and morphology regulation methods.

### 3.2 Based on MOFs’ Unique Porosity and Large BET

#### 3.2.1 Loading Guest Molecules (Cocatalysts and Photosensitizers)

**Figure 18:**
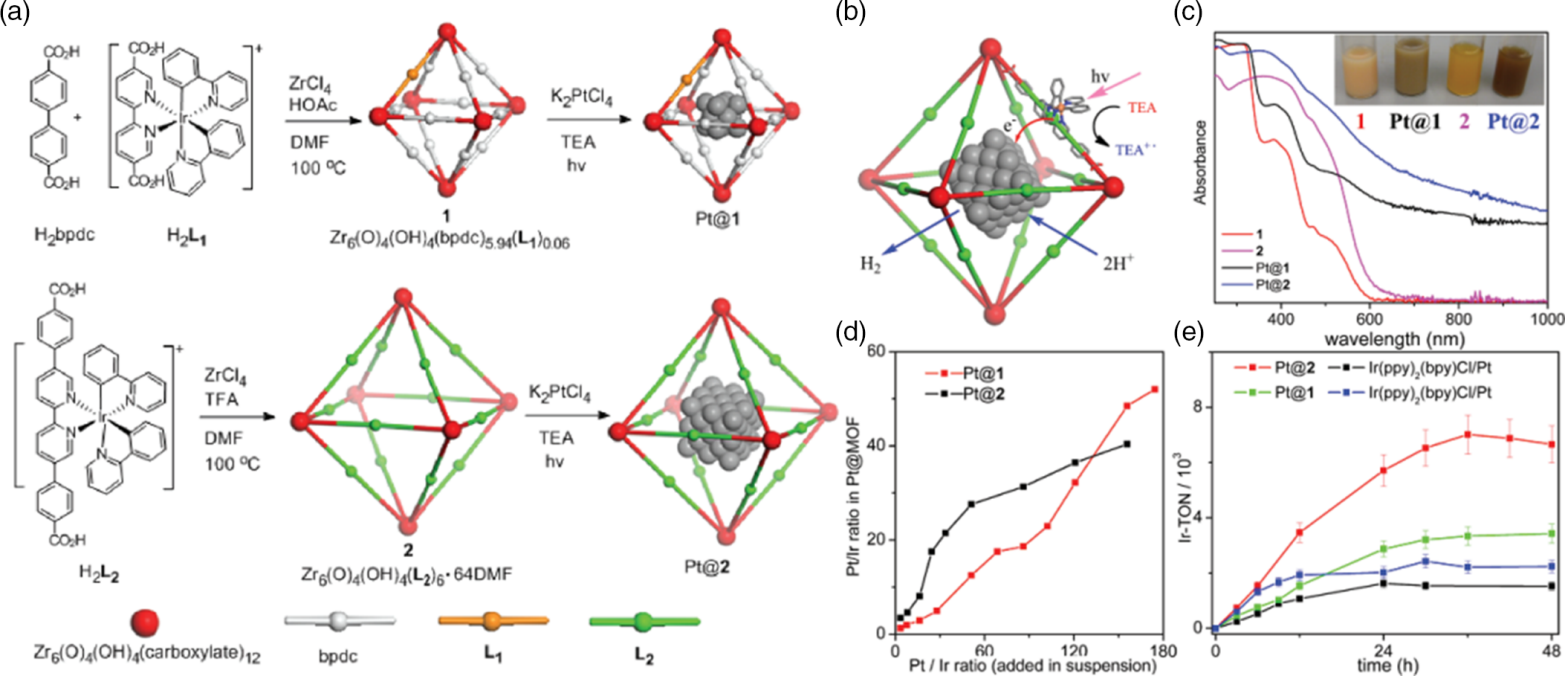
a) Synthesis of phosphorescent Zr‐carboxylate MOFs (1 and 2) of the fcu topology and subsequent loading of Pt NPs inside MOF cavities via MOF‐mediated photoreduction of K_2_PtCl_4_ to form the Pt@1 and Pt@2 assemblies. b) Scheme showing the synergistic photocatalytic hydrogen evolution process via photoinjection of electrons from the light‐harvesting MOF frameworks into the Pt NPs. The red balls represent Zr_6_(O)_4_(OH)_4_(carboxylate)_12_ cores, whereas the green balls represent the Ir‐phosphor ligand of the MOF. c) Diffuse reflectance spectra of 1 (red), Pt@1 (black), 2 (purple), and Pt@2 (blue). A photograph of suspensions of these samples is shown in the inset. d) Relationship between the amount of K_2_PtCl_4_ added in the reaction solution and the amount of Pt deposited inside the MOF (normalized to the amount of) for Pt@1 (red) and Pt@2 (black). e) Time‐dependent hydrogen evolution curves of Pt@1 (green), Pt@2 (red), and homogeneous control [Ir(ppy)_2_(bpy)]Cl/K_2_PtCl_4_ (blue and black for different Pt/Ir ratios) under optimized conditions (Pt/Ir ratios in solution/ suspension for Pt@1 and its homogeneous control is 86.0; Pt/Ir ratios in solution/suspension for Pt@2 and its homogeneous control is 24.2; stirring rate for all reactions was 1000 rpm). a–e) Reproduced with permission.^[^ ^102^ ^]^ Copyright 2012, American Chemical Society.

Due to the narrowed light absorption region and poor charge carrier separation/migration performance, most catalysts still display inefficient performance for photocatalytic H_2_ production. To solve the aforementioned problems, loading guest molecules, including cocatalysts and photosensitizers, have usually been used and been considered as an effective method for enhancing the light harvesting and charge carrier separation/migration performance of the photocatalysts. However, traditional inorganic semiconductor photocatalysts are nonporous with low specific surface area, which results in the fact that the cocatalysts or photosensitizers can only be loaded on their surface with a low concentration. In this regard, MOF‐based materials, which possess high specific surface area, have been regarded as good candidates for loading guest molecules to optimize their photocatalytic performance for H_2_ production. Noble metal NPs, such as Au, Ag, Pt, and Pd, have strong spectral absorption in the UV and visible region because of their surface plasmon resonance effect. Meanwhile, the noble metal NPs can be regarded as effective electron‐trapping sites and are beneficial for the charge carrier separation. Therefore, noble metals were usually applied as efficient H_2_ evolution cocatalysts. Especially, Pt was widely used to combine with MOF‐based materials to achieve high‐efficiency water‐splitting activity for hydrogen production. For instance, through a novel photodeposition reduction reaction of potassium cyanide platinate and UiO MOFs particles, which were built from Ir‐phosphor‐derived linear dicarboxylate linkers and Zr_6_(*μ*_3_‐O_)4_(μ_3_‐OH)_4_(carboxylate)_12_ SBUs, Pt metal NPs‐modified UiO MOFs were successfully constructed by Lin and coworkers (**Figure** **18**).^[^ ^102^ ^]^ In comparison with pristine MOFs, the prepared Pt@MOF displayed a higher conversion number (7000) and photochemical quantum yield (5.6 ± 0.4) × 10^−4^) for photocatalytic water reduction, which is due to the efficient transfer of electrons from unstable [Ir^III^(ppy)_2_(bpy^−^)] species to Pt NPs in MOFs. Through different synthesized methods, the noble metals can locate in/on different positions of the MOF‐based materials. However, the investigation on the influence of the position of noble metals for determining their photocatalytic activity has rarely been reported, especially in MOF‐based materials. To break through this bottleneck, Jiang's group incorporated Pt NPs with about 3 nm into or on a classical Zr‐MOF, UiO‐66‐NH_2_, and successfully constructed Pt@UiO‐66‐NH_2_ and Pt/UiO‐66‐NH_2_, respectively.^[^ ^54^ ^]^ Due to the presence of effective electron acceptors, both Pt‐decorated UiO‐66‐NH_2_ displayed a higher photocatalytic activity for H_2_ evolution.

Interestingly, Pt@UiO‐66‐NH_2_ exhibited a higher photocatalytic performance than Pt/UiO‐66‐NH_2_. To explain the involved mechanism, the ultrafast TA and PL spectroscopy was applied to investigate their carrier lifetime. Compared with Pt/UiO‐66‐NH_2_, the path of electron migration in Pt@UiO‐66‐NH_2_ was much shorter, thus exhibiting a higher efficiency for electron–hole pairs separation. This study provides a deeper understanding of the location of decorated noble metals for determining their photocatalytic activity for water reduction. Inspired by above, through integrating the plasmonic effect and a Schottky junction into MIL‐125, Jiang and coworkers designed the Pt@MIL‐125/Au and Pt/MIL‐125/Au ternary composite photocatalysts and continued to study the influence of the position of Pt on their photocatalytic activity.^[^ ^103^ ^]^ Similar to the results reported in previous articles, Pt@MIL‐125/Au showed the strongest photocatalytic performance. The remarkable photocatalytic activity was attributed to the surface plasmon resonance effect of Au nanorods and the Schottky junction between Pt and MOFs, which can broaden the photoresponse range of MOFs from UV light to the visible light and accelerate the separation of photogenerated carriers.

In addition to Pt, Au NPs are also widely used to improve the photocatalytic activity of MOF materials. For example, Jiang et al. first designed and reported a UCNPs‐Pt@UiO‐66NH_2_/Au complex (UCNPs denote NaYF4: Yb, Tm, Er), which can be simultaneously responsive to UV light, visible, and near‐infrared light.^[^ ^104^ ^]^ During the synthesis process, the Pt NPs were first dispersed on the surface of UCNPs, and then through a layer‐by‐layer assembly strategy, the UiO‐66NH_2_ shell can coat their surface. Finally, the Au NPs were stabilized on UiO‐66NH_2_, thus forming the resulting complex, which can spatially separate the Au and Pt NPs in one MOF material. Interestingly, in this complex, the UiO‐66NH_2_ can absorb UV light, Au NPs can be responsive to visible light due to the plasmatic effect, and the UCNPs can convert NIR light to UV and visible light due to the upconversion effect. Therefore, the resulting complex can achieve harvesting solar spectrum from UV to near‐infrared region. Benefitting from the elaborate design, the UCNPs‐Pt@UiO‐66NH_2_/Au displayed excellent photocatalytic activity for water reduction, in which the H_2_ production rate can achieve 280 μmol g^−1^ h^−1^ under simulated solar light. Cheng and coworkers designed a new type of photocatalytic material Au@UiOS@ZIS, in which Au nanodots were anchored in the pores of the sulfhydryl‐modified UiO‐66 (abbreviated as UiOS) MOF and ZnIn_2_S_4_ (abbreviated as ZIS).^[^ ^105^ ^]^ The nanosheets were wrapped around the sulfhydryl UiO‐66. As revealed by the energy dispersive spectrometer (EDS) and transmission electron microscope (TEM) results, Au NPs with an average size of about 1 nm were uniformly distributed in the pores of the MOFs and then were perfectly coated by ZnIn_2_S_4._ The study found that Au nanodots in different positions played an important role in the height of Schottky barrier (Φ_b_). For the encapsulated Au NDs in the pores of UiOS, the photocatalytic hydrogen production rate of the optimal catalyst under visible light reached 39.2 mmol g^−1^ h^−1^, which was attributed to the effective electron transfer from ZnIn_2_S_4_ to UiOS and then to Au.

**Figure 19:**
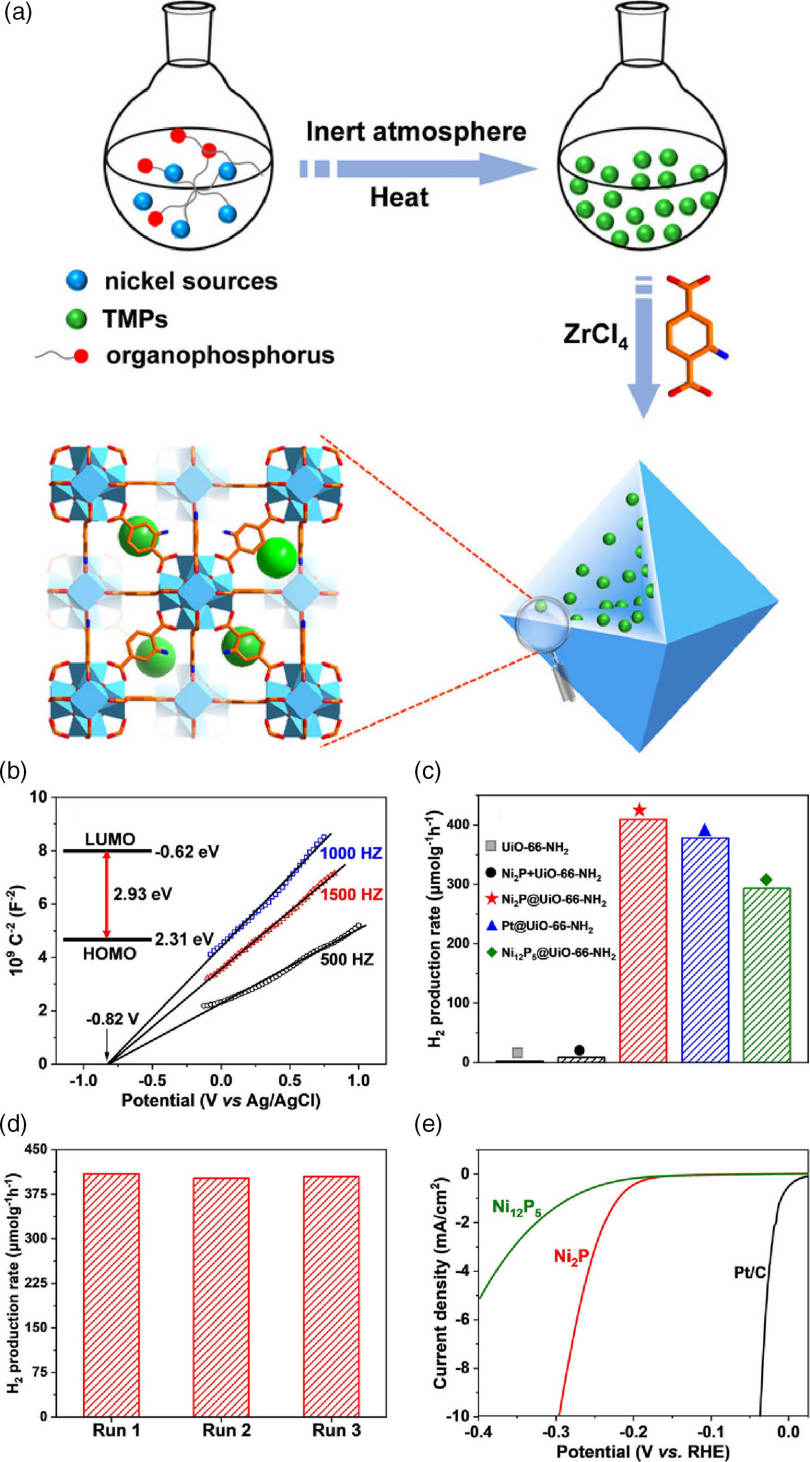
a) Schematic illustration for the synthesis of TMPs and TMPs@UiO‐66‐NH_2_. b) Mott−Schottky plots of UiO‐66‐NH_2_ in 0.1 M Na_2_SO_4_ aqueous solution (pH: 6.8) (inset: the energy diagram of UiO‐66‐NH_2_). c) The photocatalytic hydrogen production rates of UiO‐66‐NH_2_, physical mixture of Ni_2_P and UiO‐66‐NH_2_, Ni_2_P@UiO‐66‐NH_2_, Pt@UiO‐66‐NH_2_, and Ni_12_P_5_@UiO‐66‐NH_2_. d) Recycling performance of Ni_2_P@UiO‐66‐NH_2_. e) LSV curves of Ni_2_P, Ni_12_P_5_, and Pt/C. a–e) Reproduced with permission.^[^ ^107^ ^]^ Copyright 2020, Wiley‐VCH.

However, due to the high price of precious metals, seeking for noble‐metal‐free material as a cocatalyst has become a hot research direction. Among them, the use of transitional metal phosphides (TMP)‐based cocatalysts to modify MOFs to improve their photocatalytic hydrogen production reaction activity is common. For example, Ma and coworkers designed and manufactured a g‐C_3_N_4_/UiO‐66/Ni_2_P composite through a facile hydrothermal method and then compared the hydrogen production activity under visible light with g‐C_3_N_4_/UiO‐66 and g‐C_3_N_4_/Ni_2_P.^[^ ^106^ ^]^ PC, impedance, and linear sweep voltammetry (LSV) all confirmed that it had a stronger PC intensity and a more accelerated photogenerated electron–hole separation ability after the introduction of Ni_2_P as a cocatalyst. Finally, a possible reaction mechanism was proposed. Another fascinating example was reported by Jiang's group. In their work, the TMP, for example, Ni_2_P, Ni_12_P_5_, with nanoscale, was monodispersed embedded in the classical UiO‐66‐NH_2_ material.^[^ ^107^ ^]^ The incorporated TMP can play the role of cocatalysts, which can accelerate the charge carrier separation/migration, thus improving the photocatalytic activity for H_2_ evolution. The authors also proved that the location of TMP plays an important role in determining their photocatalytic activity, in which TMP@UiO‐66‐NH_2_ displayed a higher photocatalytic activity than TMP/UiO‐66‐NH_2_ because of a shorter electron transfer path. Moreover, the activity of Ni_2_P@UiO‐66‐NH_2_ even surpassed Pt@UiO‐66‐NH_2_, which can explain that Pt was thermodynamically favorable, as revealed by the energy‐level and LSV analysis, but TMP was kinetically preferred, as demonstrated by the photoelectrochemical tests (**Figure** **19**). Therefore, the TMP‐based materials have been considered as ideal cocatalysts for substituting Pt for being applied in the field of photocatalytic H_2_ production. Mxene, as a promising class of thin‐layered materials, is also an excellent cocatalyst for photocatalytic hydrogen production.^[^ 108, 109, 110 ^]^ Huang's group successfully fabricated Ti_3_C_2_@MIL‐NH_2_ composite using the in situ growth strategy. Then, the effect of Ti_3_C_2_ as a cocatalyst on the photocatalytic hydrogen production activity was systematically studied.^[^ ^111^ ^]^ In comparison with pristine MIL‐NH_2_, Ti_3_C_2_@MIL‐NH_2_ composite has not only faster electron transfer performance, but also higher photogenerated electron–hole pair separation efficiency. Therefore, Ti_3_C_2_@MIL‐NH_2_ exhibited higher photocatalytic performance for hydrogen production, in which the H_2_ evolution rate reached up to 4383.1 μmol h^−1 ^g^−1^.

In addition to overloading NPs, loading dye sensitization is also a very effective strategy to improve the photocatalytic activity of MOF‐based photocatalysts. For example, Xue and coworkers used Erythrosin B (ErB) dye as a photosensitizer to sensitize UiO‐66 octahedrons.^[^ ^112^ ^]^ Due to noncovalent *π−π* stacking and Van der Waals interactions, ErB dye molecules and UiO‐66 achieved close contact. The results indicated that the increase in ErB addition led to the increase in dye molecules on the MOF surface and can not only improve their light‐harvesting performance, but also promote the photogenerated electrons from ErB dye transfer to UiO‐66, thus achieving an efficient hydrogen production rate.

Interestingly, the coupling mode of the dye and the carrier also played an important role in determining the photocatalytic activity for hydrogen production. Guo and coworkers designed two coupling modes of Eosin Y (EY)‐sensitized UiO‐66‐NH_2_ through a room‐temperature treatment process and a hydrothermal process, respectively.^[^ ^113^ ^]^ In comparison with the room‐temperature treatment method, EY and UiO‐66‐NH_2_ could establish strong interaction (chemical bond) via the bidentate bridging configuration of Zrmi—C═Ocou through the hydrothermal process, which can load more EY molecules to promote the visible light absorption capacity, accelerate the photogenerated carrier transfer, and improve the stability of the catalyst, thus finally leading to a higher photocatalytic activity, in which the AQE at 500 nm for H_2_ evolution can reach up to 17.6%. Apart from the Eosin Y dye, the low‐cost cone‐calixarene‐based dye (Calix‐3) has also recently received tremendous attention because of its cone conformation, four light‐harvesting units per molecule and four −COOH groups in one molecule, which are beneficial to impede dye aggregation and accelerate the transmission of photogenerated carriers. Such molecules can form a strong hydrogen bond with the MOF material and then increase their stability. For example, Su and coworkers successfully designed a cone‐calixarene dye Calix‐3‐sensitized Pt@UiO‐66‐NH_2_ complex, which was then used in the photocatalytic hydrogen production reaction.^[^ ^114^ ^]^ Due to high specific surface area and functional framework of the UiO‐66‐NH_2_, Calix‐3 can uniformly be decorated on their framework, thus preventing aggregation. The resulting material can exhibit higher molar absorption coefficients and efficient electron transfer performance. Therefore, as revealed by the photocatalytic measurements, Calix‐3/Pt@UiO‐66‐NH_2_ exhibited a commendable photocatalytic hydrogen production rate of 1528 μmol g^−1 ^h^−1^. Therefore, due to the porous structure and abundant modifiable and metal coordination sites in MOF materials, loading guest molecules has been considered as one of the most effective methods for improving their photocatalytic activity for water splitting.

#### 3.2.2 Constructing Heterostructures

In addition to the use of metal modification and dye sensitization strategies, coupling MOFs with other narrow‐bandgap semiconductors, and then using the synergistic effect of heterogeneous substances, is also a seemingly good method to broaden the visible light response range and improve the photogenerated carriers’ separation ability of MOF‐based photocatalysts.^[^ 115, 116, 117, 118 ^]^ Generally speaking, in the construction of MOF heterostructures, Schottky junction, Type‐I, Type‐II, and Z‐scheme heterojunctions are mainly used to improve the separation efficiency of photogenerated carriers in photocatalysts.

MOF‐based Schottky heterojunction refers to the combination of metal or noble metal with MOFs materials and is considered as an effective strategy to improve their carrier separation efficiency. When they are in close contact with each other, electrons generally transfer from the CB of MOF‐based catalytic materials to the metal or precious metal for hydrogen production reaction, and the remaining holes participate in oxygen production reaction. In a word, the construction of this kind of heterojunction can effectively capture electrons and holes and finally improve the photocatalytic hydrogen production efficiency.^[^ ^119^ ^]^ The formation of the Schottky junction between noble metals and MOFs has been described in detail in the modification of guest molecules in Section 3.2.1 and will not be repeated here.

Type‐I heterojunction means the fact that the VB position of one semiconductor is more positive than the VB position of another semiconductor, whereas the CB position is more negative. Both electrons and holes can therefore be transferred from one semiconductor to the other, resulting in more photogenerated carriers. However, the disadvantage of this type of heterojunction is that the photogenerated carriers are concentrated in one semiconductor and easily recombined.^[^ ^119^ ^]^ In contrast, the type‐II heterojunction perfectly avoids the disadvantages of the type‐I heterojunction, which effectively inhibits the recombination of electrons and holes. Type‐II heterojunction is explained in detail as follows. The VB potential and CB potential of one semiconductor are more positive than those of another semiconductor. In this way, electrons with more negative CB potential in the semiconductor and holes with more positive VB potential are separated from each other and transferred to another semiconductor, which significantly enhance the separation of photogenerated carriers.^[^ ^119^ ^]^ For example, compared with other photocatalysts, the graphite‐phase carbon nitride g‐C_3_N_4_ with a unique structure has many advantages, including, visible light responsiveness, nontoxicity, stability, being rich in sources, and being simple in preparation. Therefore, it has been often used to form a type‐II heterojunction with MOF photocatalysts and finally achieves efficient visible light water splitting. Recently, a binary compound g‐C_3_N_4_/UiO‐66 was rationally designed by annealing a mixture of g‐C_3_N_4_ and UiO‐66 under argon.^[^ ^120^ ^]^ Due to the enhanced visible light response and the effective electron transfer from g‐C_3_N_4_ to UiO‐66, the binary compound g‐C_3_N_4_/UiO‐66 exhibited outstanding photocatalytic activity for H_2_ evolution (0.82 × 10^−6^ M h^−1^). Subsequently, Yue and coworkers used a simple electrostatic self‐assembly strategy to coat positively charged UNiMOFs on the surface of negatively charged g‐C_3_N_4_ and designed a novel 2D/2D heterostructured photocatalyst, named UNiMOFs/g‐C_3_N_4_.^[^ ^121^ ^]^ TEM images and XPS results conclusively confirmed the successful formation of the heterojunction between g‐C_3_N_4_ and UNiMOFs. As demonstrated by the photocatalytic activity tests, the amount of UNiMOFs in the complex can significantly affect their photocatalytic activity. The UNG‐25.0 catalyst, which was composed of 100 wt% MOFs and 25 wt% g‐C_3_N_4_ with the optimal mixing ratio, displayed the best hydrogen production capacity of 20.03 μmol h^−1^. In virtues of the proper energy‐level arrangement, the synergistic effect between UNiMOFs and g‐C_3_N_4_ can shorten the transmission distance of photogenerated carriers and suppress the recombination rate under visible light irradiation. Electrons can be transferred from g‐C_3_N_4_ to UNiMOFs, and then with the help of the unsaturated Ni sites provided by UNiMOFs, hydrogen ions can be efficiently reduced to hydrogen. In addition, because of the narrowed bandgap and excellent photocatalytic ability, CdS has also been considered as good candidate for being applied in the field of photocatalytic water reduction.^[^ 122, 123, 124 ^]^ However, the fast recombination of electron–hole pairs and the few catalytic sites restrain their photocatalytic activity. Benefitting from the porous framework and high BET surface area, MOFs have been widely used to integrate with CdS to form a type‐II semiconductor heterojunction, thus improving their photocatalytic activity. For instance, Cui's group assembled CdS NPs on hollow Ni‐based MOF (Ni‐MOF) spheres through a simple in situ growth method and finally formed a novel noble‐metal‐free CdS/Ni‐MOF heterojunction.^[^ ^125^ ^]^ The close contact between CdS and NH_2_‐MIL‐125(Ti) in the heterojunction has a positive effect on charge separation. Therefore, the best CdS/Ni‐MOF(40) nanocomposite (where 40 referred to the mass ratio of CdS in the composite) had excellent photocatalytic performance of 2508 μmol g^−1 ^h^−1^ under visible light irradiation, which was eight times that of the original CdS. At the same time, their high stability can be confirmed by a cyclic test, in which only a slight decrease in activity can be observed. Apart from CdS, a variety of transition metal sulfides have also been used to form heterojunctions with MOF‐based catalysts to improve photocatalytic activity. Zn_0.2_Cd_0.8_S, as a semiconductor photocatalyst with strong visible light response and stable properties, has always been incorporated with MOFs materials to construct efficient heterojunctions for promoting H_2_ evolution. Wang and coworkers successfully constructed a new hybridization photocatalyst by coupling amino‐functionalized Zr‐based MOFs (UiO‐66‐NH_2_) and Cd_*x*_Zn_1−*x*_S.^[^ ^126^ ^]^ The super visible light‐response capability of Cd_*x*_Zn_1−*x*_S and the accelerated photogenerated carrier transfer after forming a heterojunction helped to improve their photocatalytic activity. As a result, such a heterojunction displayed high hydrogen production rate of 5846.5 μmol h^−1 ^g^−1^, which was equal to 2.1 times that of pure Cd_*x*_Zn_1−*x*_S. After four cycles, the photocatalytic hydrogen production activity of the MOF composite catalyst hardly decreased, which further indicated that the construction of a heterogeneous structure was beneficial to enhance the stability of the photocatalyst. Subsequently, Qiao et al. used 2D MOF [Ni(phen)(oba)]*n*·0.5*n*H_2_O as a multifunctional substrate to support CdS or Zn_0.8_Cd_0.2_S and constructed an efficient composite photocatalyst.^[^ ^127^ ^]^ The synchrotron‐based X‐ray absorption near‐edge structure and theoretical calculations confirmed the strong electronic coupling between CdS and 2D NMF. Coupling the large surface area, highly exposed active sites, and suitable energy band structure, the photocatalytic heterojunction system exhibited excellent photocatalytic performance. When lactic acid was used as a sacrificial agent and the promoter Pt was not used, it still showed an ultrahigh photocatalytic hydrogen production capacity of 45 201 μmol h^−1 ^g^−1^. In addition, ZnIn_2_S_4_ is a ternary chalcogenide which has a suitable bandgap (2.34−2.48 eV) and can respond well to visible light. It has been reported that combining it with MOFs catalysts to construct heterostructures can improve their photocatalytic performance. Ao and coworkers reported a series of ZnIn_2_S_4_@NH_2_‐MIL‐125(Ti) photocatalytic heterojunction systems, which were composed of aminated MIL‐125(Ti) and different amounts of ZnIn_2_S_4_.^[^ ^128^ ^]^ The photoelectrochemical tests proved that the heterojunction had a strong ability to generate and transfer the photoexcited electron–hole pairs due to the synergy effect between ZnIn_2_S_4_ and NH_2_‐MIL‐125(Ti). Benefiting from these advantages, the photoexcited electrons on the CB of ZnIn_2_S_4_ can rapidly transfer to the LUMO of NH_2_‐MIL‐125(Ti) and then efficiently convert hydrogen ions into hydrogen on the catalyst surface under visible light illumination. More recently, the combination of TiO_2_ photocatalytic materials and MOF‐based photocatalytic materials to improve the activity of photocatalytic hydrogen production has received great attention. For example, Han and coworkers synthesized the TiO_2_@NH_2_‐MIL‐125(Ti) heterostructure with different degrees of exposed active crystal planes by etching.^[^ ^129^ ^]^ The composite photocatalyst had a tight heterojunction interface and open diffusion channels because the Ti species of TiO_2_ in the formed TiO_2_@MOF frame structure (FS) came from NH_2_‐MIL‐125 (Ti). Compared with the pure catalyst, TiO_2_@NH_2_‐MIL‐125(Ti) with exposed [100] surface showed a higher photocatalytic reduction of water to produce hydrogen. Such high photocatalytic activity came from high conductivity and open diffusion channels for photogenerated carriers, which can be confirmed by PC, impedance, and LSV curves.

**Figure 20:**
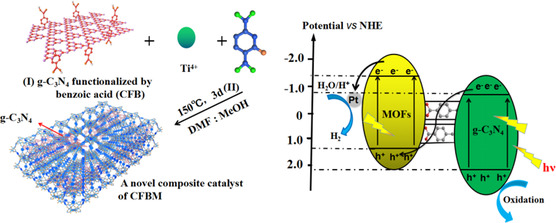
Schematic representation of the synthesis of covalently linked CFBM composites. I) g‐C_3_N_4_ functionalized by benzoic acid (CFB) and II) in situ growth of CFBM crystals and the photocatalytic mechanism of the charge transfer for hydrogen evolution over 10CFBM under visible light irradiation. Reproduced with permission.^[^ ^130^ ^]^ Copyright 2018, Elsevier.

However, the disadvantage of type‐II heterojunction is that it can only use the relatively low VB position and the relatively high CB position in the heterojunction. Therefore, to further improve the separation efficiency of photogenerated carriers in photocatalysts, a new Z‐scheme heterojunction was proposed. Z‐Scheme heterojunction means that after two kinds of semiconductors form heterojunction with each other, holes with lower VB potential and electrons with higher CB potential in the two semiconductors will directly recombine under the irradiation of visible light and UV light, leaving VB with strong oxidation ability and CB with strong reduction ability in the composite catalyst, thereby utilizing electrons and holes to the maximum extent and obviously improving the photocatalytic efficiency.^[^ ^119^ ^]^ For instance, Zhang et al. designed a novel Z‐type heterojunction CFB/NH_2_‐MIL‐125(Ti) (CFBM), which is tightly connected through covalent bonds.^[^ ^130^ ^]^ The benzoic acid functional group could not only establish strong interaction between carbon nitride and NH_2_‐MIL‐125(Ti), but could also build the electron transmission channel between two materials, thus inhibiting the electron–hole pair recombination. The successful construction of Z‐scheme heterojunction can be proved by the UV–vis diffuse reflection spectra (DRS) and Mott–Schottky plots. The Z‐scheme heterojunction can accelerate the separation of photogenerated carriers in CFB/NH_2_‐MIL‐125(Ti) and help to obtain excellent photocatalytic hydrogen production activity (**Figure** **20**).

**Figure 21:**
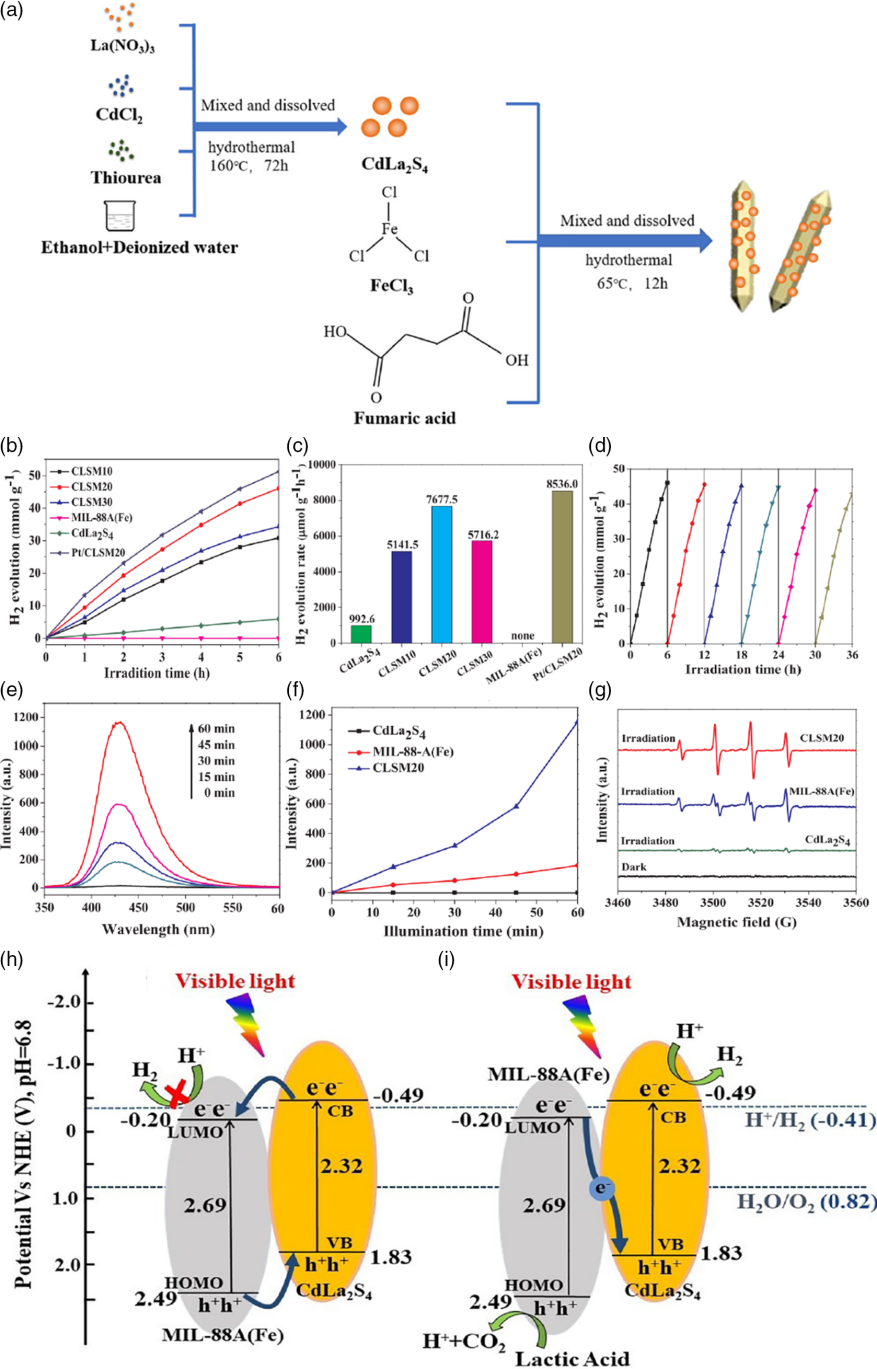
a) Synthesis process of CdLa_2_S_4_/MIL‐88 A(Fe) heterojunctions. b)Time‐dependent photocatalytic H_2_ evolution over the different samples and c) H_2_ evolution rates of the different samples. d) Cycling runs for photocatalytic H_2_ evolution over CLSM20 under visible light. e) OH‐trapping PL spectra of the CLSM20 sample; f) comparison of PL intensity at 430 nm for CdLa_2_S_4_, MIL‐88 A(Fe), and CLSM20. g) DMPO spin‐trapping ESR spectra of CdLa_2_S_4_, MIL‐88 A(Fe), and CLSM20 recorded in an aqueous dispersion (for DMPO − OH) by irradiation with visible light. Possible electron transfer mechanism over CdLa_2_S_4_/MIL‐88 A(Fe): h) type‐II heterojunction and i) direct Z‐scheme. a–i) Reproduced with permission.^[^ ^133^ ^]^ Copyright 2020, Elsevier.

Due to the ideal bandgap position (2.1−2.5 eV) and appropriate CB position, CdLa_2_S_4_ is a kind of promising hydrogen production photocatalyst. However, due to its severe photogenerated carrier recombination rate, the photocatalytic activity is often not excellent. Using CdLa_2_S_4_ to combine with other semiconductors to form an appropriate energy band structure has proven to be a very effective method to avoid this disadvantage.^[^ 131, 132 ^]^ Liu et al. used a one‐step hydrothermal method to introduce CdLa_2_S_4_ into MIL‐88 A(Fe) and constructed a Z‐Scheme CdLa_2_S_4_/MIL‐88 A(Fe) photocatalytic system.^[^ ^133^ ^]^ As expected, the maximum light absorption edge of CdLa_2_S_4_/MIL‐88 A(Fe) had a red shift due to the mixed absorption characteristics of single MIL‐88 A(Fe) and CdLa_2_S_4_. In addition, when the content of MIL‐88 A(Fe) was up to 20 wt%, the optimal CdLa_2_S_4_/MIL‐88 A(Fe) heterojunction photocatalytic hydrogen production (7677.5 μmol g^−1 ^h^−1^) was about 7.73 times that of pure CdLa_2_S_4_. Furthermore, electron spin‐resonance spectroscopy (ESR) was used to confirm that the mechanism of photogenerated carrier transfer following the Z‐Scheme mechanism rather than the type‐II mechanism (**Figure** **21**).

Herein, benefitting from the high specific surface area, MOF materials can provide an ideal platform for integrating with other materials with strong interaction and constructing heterojunctions to improve their photocatalytic activity for water reduction.

#### 3.2.3 MOFs as Templates to Construct Porous Traditional Semiconductors

MOF derivatives, which are constructed using MOFs as precursors through a calcination process, have been widely studied. The resultant materials could not only inherit the porous structure of MOFs, but also have higher electronic conductivity than MOF materials. Therefore, such kinds of photocatalysts display higher carrier transfer efficiency, thus receiving great attention in recent years. Due to the advantages of porous structure and easy preparation, ZIFs, a kind of stable MOF material, have been widely used as sacrifice templates to construct MOF derivative photocatalyst, including metal oxide, metal sulfide, metal phosphide, g‐C_3_N_4_, and their composite photocatalysts.

**Figure 22:**
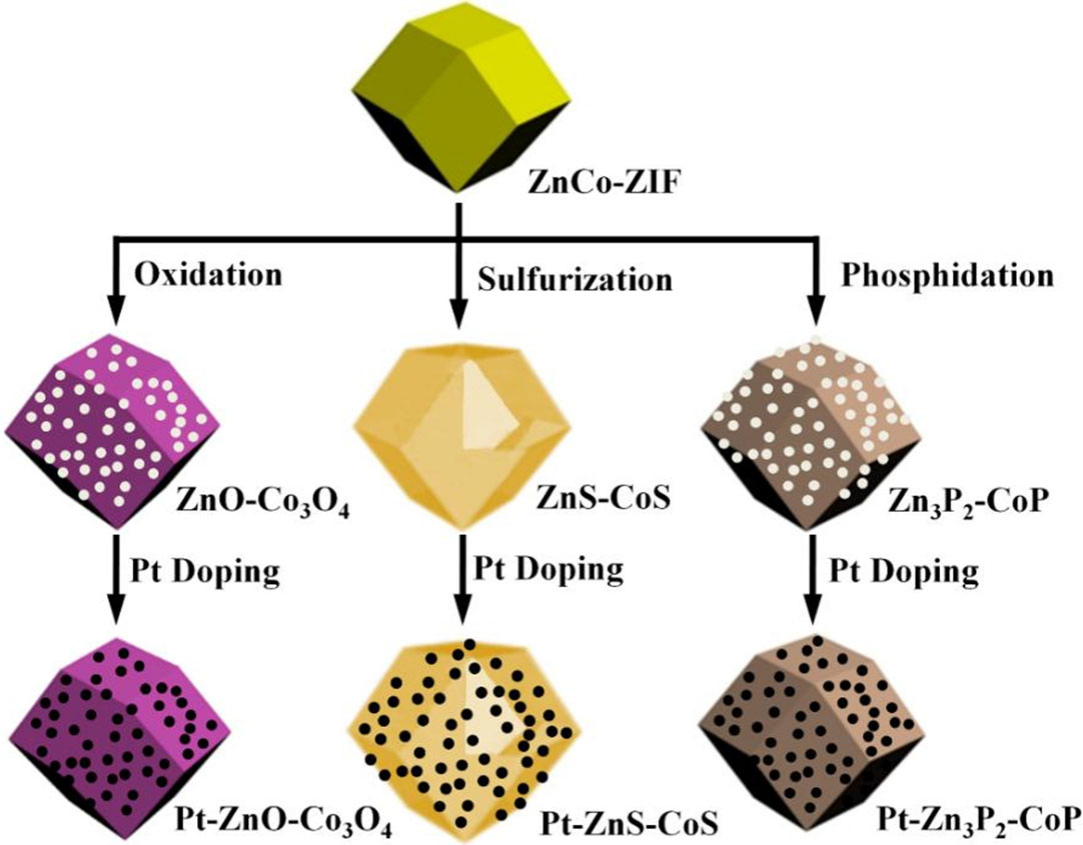
The schematic illustration of the fabrication of Pt–ZnO–Co_3_O_4_, Pt–ZnS–CoS, and Pt–Zn_3_P_2_–CoP photocatalysts. Reproduced with permission.^[^ ^134^ ^]^ Copyright 2017, Elsevier.

For example, Cheng et al. used ZIF‐8 as precursor and then prepared nitrogen‐doped graphene analogs through a one‐step calcination method and then used it for photocatalytic H_2_ production.^[^ ^68^ ^]^ Interestingly, the nitrogen content and even the nitrogen type of the product can be changed through changing the calcination temperature. The nitrogen content and type are also closely related to their photocatalytic activity. The experimental results confirmed that when the temperature was 1000 °C, the product was completely graphite nitrogen and displayed the highest photocatalytic hydrogen production performance, which came from the high mobility of photogenerated carriers. Moreover, Li and coworkers synthesized a series of metal oxide/sulfide/phosphide heterojunctions through oxidation, sulfurization, and phosphorization of ZnCo‐ZIF, respectively (**Figure** **22**).^[^ ^134^ ^]^ Benefitting from the huge surface area of the MOF precursor, their derivatives can maintain the porous structure, which can not only improve their light‐harvesting performance, but also enrich their reactive sites. Moreover, using the bimetallic ZIFs as templates, the heterojunctions can be constructed directly with suitable band matching and strong electronic coupling, which can facilitate the charge carrier separation and migration.

Therefore, the resulting heterojunctions, including Pt–ZnO–Co_3_O_4_, Pt–ZnS–CoS, and Pt–Zn_3_P_2_–CoP, displayed high photocatalytic activity for water reduction, in which the H_2_ production rate was about 7.80, 8.21, and 9.15 mmol h^−1^ g^−1^, respectively, which is higher than pure ZIF‐8 or ZIF‐67 derivative photocatalysts. Recently, Zhao's group used slight oxidation treatment, phosphating, and sulfidizing of Co‐based ZIF‐67 to obtain three MOF‐derived fold polyhedrons, named, O‐ZIF‐67, P‐ZIF‐67, and S‐ZIF‐67, respectively.^[^ ^135^ ^]^ Due to the hyperchromic effect, the materials all exhibited extended light responsiveness compared with the wide bandgap of Co‐based ZIF‐67. Likewise, the photocatalytic activity of fold polyhedrons can be enhanced using TEOA as sacrificial agent and EY dye as photosensitizer. Among the three materials, P‐ZIF‐67 exhibited the best photocatalytic activity, which is derived from enhanced electron capture capability and light absorption intensity, a more negative flat‐band, and smaller overpotential.

**Figure 23:**
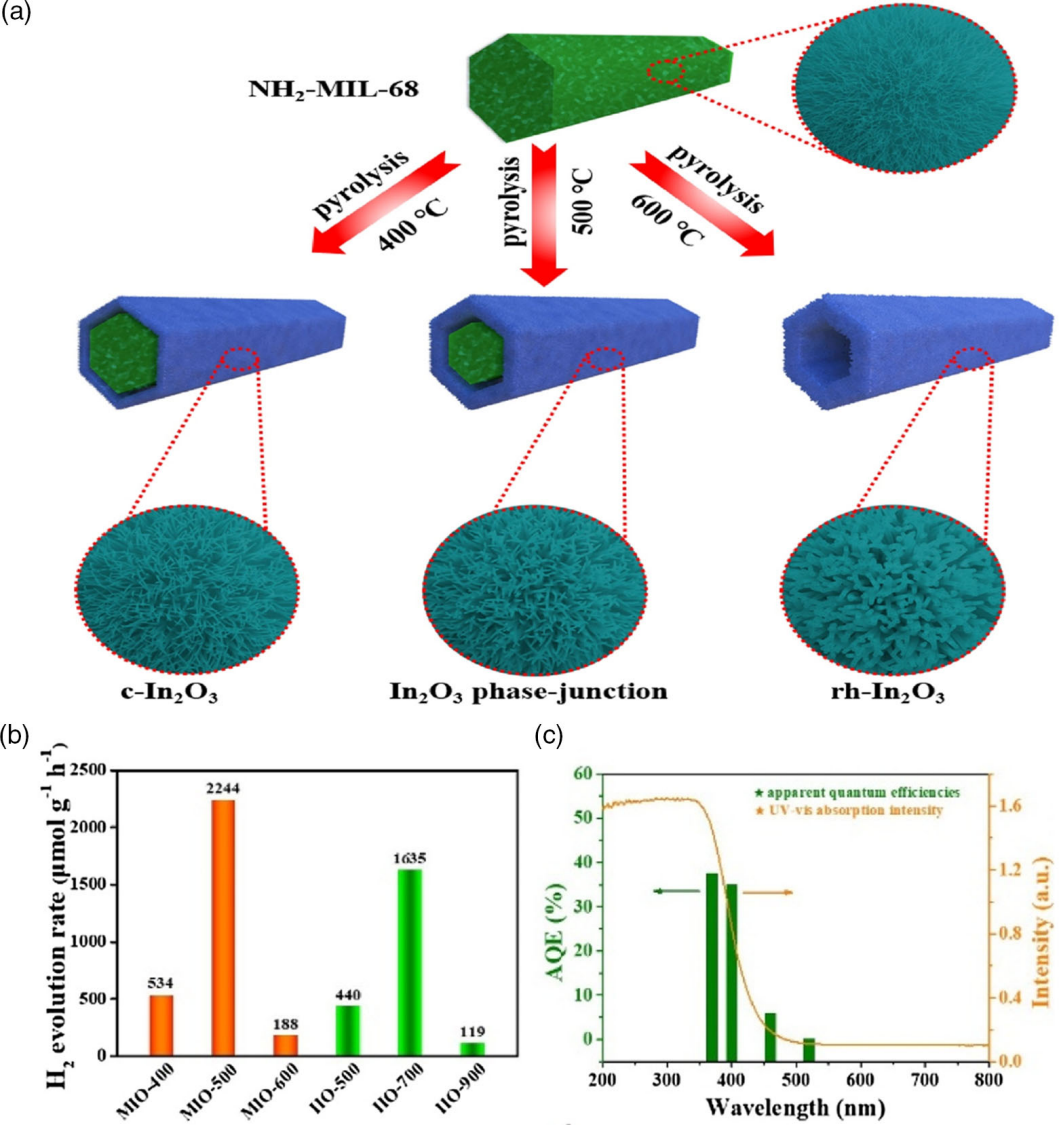
a) Schematic illustration of the synthetic process of c‐In_2_O_3_, In_2_O_3_ phase junction, and rh‐In_2_O_3_. b) Photocatalytic H_2_ evolution activities for MIO and IIO samples. c) UV–vis spectrum and AQE of MIO‐500. a–c) Reproduced with permission.^[^ ^138^ ^]^ Copyright 2021, Elsevier.

TiO_2_ is also a kind of important photocatalyst which is suitable for being applied in the field of H_2_ evolution. However, the drawbacks of few reactive sites and dense structures still have to be solved. In this regard, Ti‐based MOFs have been recognized as ideal templates to construct porous TiO_2_ photocatalysts with predesigned morphology and porous structure. For example, in 2017, a Pd‐decorated hierarchical TiO_2_ was successfully designed through pyrolysis of NH_2_‐MIL‐125(Ti) combined with photoreduction process.^[^ ^136^ ^]^ The synthesized photocatalyst can significantly broaden the visible light response capability and provides abundant reactive sites, which can be attributed to their porous framework derived from MOFs. The effective separation of photogenerated carriers enables it to be used in photocatalytic hydrogen production reactions. Using MOFs as precursor, the BiVO_4_‐based solid solution, Bi_0.5_Y_0.5_VO_4_, can also be prepared through combining solid solution calcining and hydrothermal method.^[^ ^137^ ^]^ A series of characterization methods was used to confirm the formation of multiphase BiVO_4_. Through increasing the calcining time, the amorphous Bi_0.5_Y_0.5_VO_4_ can be converted to the Bi_0.5_Y_0.5_VO_4_ with tetragonal phase, thus changing their twisting degree and influencing the internal electric field. Consequently, the BiVO_4_‐based solid solutions derived from Bi‐based MOFs were able to achieve high‐efficiency photocatalytic hydrogen production performance. Moreover, Zhou and coworkers also used the metal organic framework (MIL‐68(In)‐NH_2_) to be annealed in air for constructing the Z‐scheme hexagonal/cubic In_2_O_3_ phase junction (**Figure** **23**a).^[^ ^138^ ^]^ A variety of characterization techniques, such as the steady‐state surface photovoltage (SPV) spectra, the steady‐state PL spectrum, the TRPL decay spectra, the PC response, and EIS, has been used to explore the transfer efficiency of photogenerated carriers in the catalyst.

As expected, MIO‐500 (which refers to annealing MIL‐68(In)‐NH_2_ at the target temperature of 500 °C) has the strongest SPV response, lowest PL intensity, longest photogenerated carrier lifetime, strongest PC response, and the smallest impedance. Therefore, the recombination of photogenerated carriers is severely inhibited. At the same time, ESR was used to confirm the Z‐type‐accelerated electron transport mechanism of the phase heterojunction. Therefore, the c‐In_2_O_3_/rh‐In_2_O_3_ heterojunction achieved a high hydrogen production rate of 2244 μmol h^−1 ^g^−1^, and the quantum efficiency was about 35% at 400 nm, which can be equal to 12 times that of pure c‐In_2_O_3_ (Figure 23b‐c).

MOF materials were assembled by the metal cores and organic ligands. Therefore, in addition to constructing metal‐based photocatalysts, MOFs can also be used as precursors to build carbon‐based photocatalysts. Meanwhile, sometimes the metal source can also be reserved to form cocatalysts or heterojunctions. For instance, Liu and coworkers designed a carbon (C‐ZIF)/g‐C_3_N_4_ composite through facile thermal condensation of ZIF‐8 and melamine.^[^ ^139^ ^]^ A series of characterization methods such as PL spectroscopy, time‐resolved fluorescence spectroscopy, and EIS has been used to indicate that the construction of heterostructures can improve the separation ability of photogenerated carriers. Benefiting from this, significantly enhanced photocatalytic activity is obtained. The photocatalytic hydrogen production rate of 1 wt% C‐ZIF/g‐C_3_N_4_ composite was equal to 36 times that of carbon nitride and 2.8 times that of Pt/g‐C_3_N_4_.

**Figure 24:**
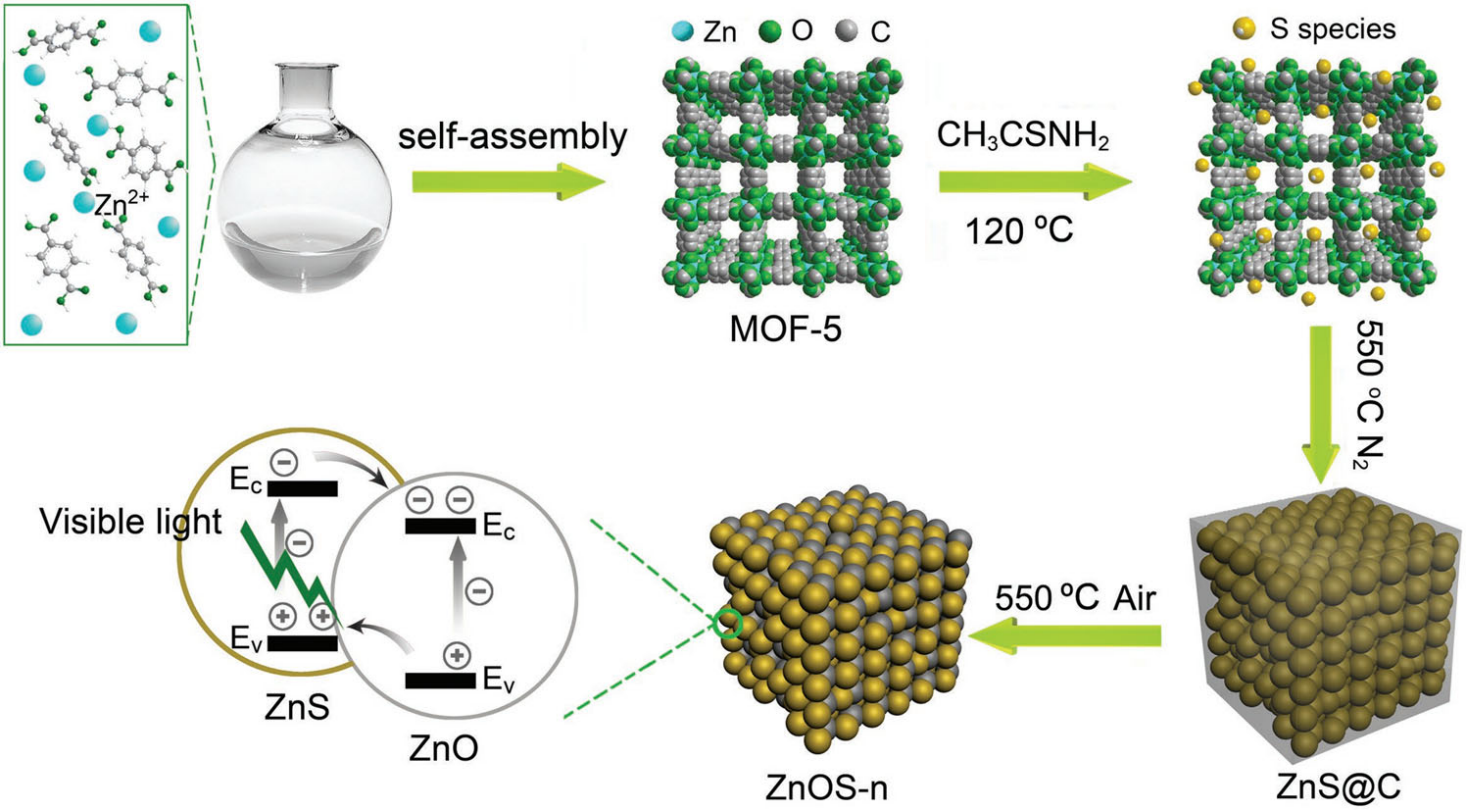
Schematic illustration of the synthetic procedure of ZnO/ZnS heterostructures. Reproduced under the terms of the CC‐BY 4.0 license.^[^ ^140^ ^]^ Copyright 2018, The Authors, published by Wiley‐VCH.

Moreover, templating method could also be used to directly prepare heterojunctions. Cheng and coworkers reported a simple and novel method to prepare a ZnO/ZnS heterojunction (**Figure** **24**).^[^ ^140^ ^]^ First, thioacetamide was used to generate hydrogen sulfide; then, it was used to calcinate MOF‐5 to generate ZnS@C at high temperature, and finally it was calcined in air to generate ZnO/ZnS heterojunction. The authors also synthesized a series of samples with different proportions by changing the calcination time.

Due to the enhanced visible light response capability and improved photogenerated carrier separation efficiency provided by the heterojunction, when Na_2_S and Na_2_SO_3_ were used as sacrificial agents, even without cocatalysts, the ZnS/ZnO mixture displayed a higher photocatalytic activity for hydrogen production (415 μmol h^−1 ^g^−1^) than the pure ZnS. In addition, the bimetallic Fe–Ni–P nanotubes can also be successfully constructed by calcining a mixture of FeNi_2_‐MIL‐88 and NaH_2_PO_2_ at 320 °C.^[^ ^141^ ^]^ The successful formation of the bimetallic Fe–Ni–P nanotubes could be confirmed by TEM and XPS results. The synthesized Fe–Ni–P nanotubes displayed better photocatalytic hydrogen and oxygen production performance compared with Fe_2_P and Ni_2_P samples. The enhanced photocatalytic activity can be attributed to the successful construction of heterostructures, which can facilitate the electron–hole pairs’ migration and separation, thus resulting in a higher photocatalytic water reduction performance. Benefitting from the high specific surface area and porous structure, MOFs materials can not only load guest molecules and be combined with other materials to construct heterojunctions, but can also be supplied as templates to prepare traditional photocatalysts, endowing them with a porous framework and abundant reactive sites.

However, due to the high price of the MOF materials, it will increase the cost of prepared materials through such methods, which have to be solved. In Table 4, we show the examples for optimizing the photocatalytic activity of MOFs for water reduction through the aforementioned three methods. We hope this will be helpful for the researchers who are interested in this field.

## 4 Conclusion

In this review, the research progress of MOF‐based photocatalysts in photocatalytic hydrogen production has been summarized from the following aspects. 1) The categories of MOF‐based photocatalysts, which include pure MOFs photocatalysts, MOF composite photocatalysts, and MOF‐derived photocatalysts, are discussed. 2) Highlighted are the strategies for optimizing the photocatalytic activity of MOF‐based photocatalysts for H_2_ evolution, including ligand functionalization, metal doping, defect control and morphology regulation (based on MOFs tailored structure), loading guest molecules, construction of heterojunctions, and MOF‐derived photocatalysts (based on MOF porous structure). MOF‐based photocatalyst has a tailored structure and large specific surface area, which provide adjustable energy band structure and abundant exposed active sites, thus supporting their high photocatalytic performance for hydrogen production. However, some obstacles still have to be faced and solved. First, due to the weak coordination bond, only few MOFs possess high chemical stability, such as UiO‐series, MIL‐series, and ZIF‐series MOFs. Most MOF structures easily collapse during the photocatalytic reactions, which restrict their further practical applications. Therefore, it is urgently required to construct more MOFs with high chemical stability. Based on previous works, two main efficient methods can be used to construct stable MOFs, including using high‐connected metal clusters as building units to construct MOFs and decorating hydrophobic substituents into the framework of MOFs. Second, introducing noble metals as cocatalysts into the MOFs is an efficient method to relieve the electron–hole pair recombination, but their high cost reduced their practical values. Therefore, it is necessary to develop some low‐cost cocatalysts, whose performance can be comparable with noble metals, thus combining with MOFs to construct efficient photocatalysts. Moreover, constructing pure MOFs photocatalysts, which do not need cocatalysts, is also an efficient but hard method to solve the above problems. Third, although a series of modification strategies for optimizing the photocatalytic activity of MOF‐based materials have been reported, most strategies focus on combining MOFs with other materials to solve their drawbacks of limited visible light response and serious recombination of charge carriers; few works concentrate on improving the activity of pristine MOFs. Therefore, more efforts should be put to optimize the physicochemical properties of the pure MOF photocatalysts through some emerging strategies, including facet regulation, defect control, and morphology regulation. Fourth, most reported MOF‐based catalysts are only used in the field of hydrogen production. Few works have been reported wherein the MOF‐based photocatalysts can produce oxygen and hydrogen simultaneously because, in terms of kinetics and thermodynamics, the overall water splitting is hard to achieve. Therefore, designing an efficient photocatalytic system based on MOFs to achieve complete water decomposition can greatly improve and avoid energy shortage. Finally, in comparison with traditional inorganic semiconductor materials, MOF‐based materials display many advantages in the photocatalytic H_2_ evolution. However, their detailed mechanisms for photocatalytic HER have not been clearly understood. Thus, combining the theoretical calculation and advanced in situ characterization method is a necessary and efficient method to explore the mechanisms of MOF‐based materials for photocatalytic water reduction with in‐depth insights.

Generally speaking, in the past ten years, with the efforts of successive researchers, MOF‐based catalytic materials have made great breakthroughs in the field of hydrogen production by photocatalytic decomposition of water, but these are far from enough, and there is still a long way to go before we expect to use solar energy to generate clean hydrogen energy and eliminate the energy crisis in the world today. The author believes that in the foreseeable future, MOF‐based materials will play a more important role in the field of photocatalytic hydrogen production. We sincerely hope that this review will be helpful to those who want to study MOF‐based catalysts for water‐splitting reactions in the future.

## Conflict of Interest

The authors declare no conflict of interest.
